# Investment expectations by vulnerable European firms in times of COVID

**DOI:** 10.1007/s40821-022-00218-z

**Published:** 2022-08-10

**Authors:** Alex Coad, Sofia Amaral-Garcia, Peter Bauer, Clemens Domnick, Peter Harasztosi, Rozália Pál, Mercedes Teruel

**Affiliations:** 1 Waseda University, Tokyo, Japan; 2 Joint Research Centre, European Commission, Brussels, Belgium; 3 Joint Research Centre, European Commission, Seville, Spain; 4 European Investment Bank, Luxembourg City, Luxembourg; 5 Department of Economics, GRIT and ECO‑SOS, Universitat Rovira i Virgili, Tarragona, Spain

**Keywords:** Investment, COVID, EIBIS, Difference-in-difference, High-growth firms, R&D investors

## Abstract

The effect of the COVID shock on European economies has been severe and also unequal, with some firms being affected much more strongly than others. To improve the effectiveness of policy interventions, policymakers need to understand which types of vulnerable firms have been suddenly pushed into dire circumstances. We seek to fill this important gap in our knowledge by providing evidence from the European Investment Bank Investment Survey 2016–2020 on how the COVID shock has affected the investment activity and investment-related framework conditions of vulnerable firms. While data on actual investment activity post-COVID is not yet available to us, we focus on investment expectations. We exploit the fact that the same questions relating to investment expectations have been asked in several previous survey waves, which enables a difference-in-differences approach to investigate how investment expectations might have suddenly changed, for vulnerable groups of firms, immediately after the onset of the COVID crisis. We focus on 4 groups of vulnerable firms: High-Growth Enterprises (HGEs), young and small firms, R&D investors and non-subsidiary firms. R&D investors are more likely to be pessimistic about investment plans as a consequence of the COVID shock, and (similarly) HGEs are less likely to be optimistic about investment plans. R&D investors are less likely to be optimistic about the availability of internal finance, while HGEs and R&D investors are more likely to be pessimistic about the availability of external finance. Subsidiary firms, interestingly, are more likely to report a decrease in expected investment, which is not necessarily evidence of financial constraints, because it could instead be part of a conservative group-level strategy and coordinated group-level reduction in investment. Event study graphs generally confirm our regression results.

**Supplementary Information:**

The online version contains supplementary material available at 10.1007/s40821-022-00218-z.

## Introduction

The COVID crisis has hit European economies in a way that is harsh and unequal. Harsh in the sense that the sudden drop in income for many businesses has been of an unprecedented magnitude. Unequal in the sense that some businesses have been pushed to the brink while others have actually been able to benefit from the COVID shock. Some sectors (such as travel agencies, accommodation, and food & beverage service activities) have seen their sales plummet in an exceptionally fast way, while other sectors such as ICT have actually benefitted (Benedetti Fasil et al., 2021; Claeys et al., 2021). Bloom et al. (2021) report that small firms with offline business models fare considerably worse than large firms with online business models, and they also find that female and black business owners faced significantly larger drops in sales, in their analysis of survey data on US firms. Overall, the literature shows that some firms have been hit much harder than others (firms in exposed sectors, small firms, firms with little online presence, firms whose owners are from minorities, etc.) and the specific needs of certain kinds of firms merits the attention of policymakers (Benedetti Fasil et al., 2021; Bloom et al., 2021; Claeys et al., 2021).

In good times, policymakers are interested in identifying and supporting various groups of vulnerable firms, which are often considered to make a disproportionately large contribution to areas such as productivity growth, job creation, and ultimately economic growth. Small firms are often targeted for policy support, because of widely-held views that small firms create jobs (Storey, 1994). Young firms are often sought out by policy makers, given that they create more jobs than small firms, and because they suffer from issues such as asymmetric information due to a lack of reputation and trading history (Coad, 2018). Innovative firms are often supported, because the social benefits of investments in innovation often exceed the short-run private returns (Biancalani et al., 2022; Mohnen & Hall, 2013). High-Growth Enterprises (HGEs) are often targeted for policy support, because of their contributions to job creation, innovation, and economic dynamism (Flachenecker et al., 2020; Mogos et al., 2021). Moreover, non-subsidiary firms are assumed to have relatively higher levels of vulnerability compared to subsidiaries, in the sense that they are not part of a larger business group such as subsidiaries, that in times of crisis, may be able to access the financial “deep pockets” of the parent company.

In bad times, the case for supporting these types of firms (*e.g.,* young and small, innovative, high-growth enterprises) becomes stronger, because the prevailing crisis may place these vulnerable firms in unparalleled difficulties. The aim of this research is to provide new evidence on the investment behavior of vulnerable groups of firms in times of COVID, using the available data on expectations surrounding investment activities. This seems like an important objective, because the sudden and unprecedented nature of the COVID shock has left policymakers in sudden need of evidence on the investment activities of vulnerable firms, while at the same time suffering from a lack of evidence on which kinds of firms are the most vulnerable.

A wide range of COVID support packages have been set up by governments to provide assistance to vulnerable firms to weather the COVID shock, such as loans, job-retention schemes, state-backed loan guarantees, debt moratoria, tax and social security deferrals and relief, securities purchases, and large-scale transfers set up to provide support and credit to firms to survive the initial “hibernation period” (Didier et al., 2021; Harasztosi et al., 2022). Naturally, there are concerns about whether these emergency support packages are reaching the firms that need them the most (Cirera et al., 2021; Harasztosi et al., 2022; Lalinsky & Pál, 2021). There are also the usual concerns about possible ‘government failures’ according to which public policy initiatives may not be attaining their goals in a cost-effective way—with resources either being allocated to artificial life support for undeserving low-performing firms (the “substitution effect”), or resources being allocated to high-performing firms who would have thrived even in the absence of these resources (the “deadweight effect”; Vivarelli, 2016). Bighelli et al. (2021) investigate the matter for 4 European countries (Croatia, Finland, Slovakia, and Slovenia). They observe that, first of all, COVID support reached mainly medium productive firms, which is good news because it is neither being given to high-productivity firms (who will survive without it) or to low-productivity firms (who may be less ‘deserving’ of support). Second, they observe that more productive firms received lower relative size of the support, which is also encouraging because they presumably have lesser needs. Third, growing firms received more support, while only a small share of support (such as wage subsidies) went to ‘zombies’ (financially distressed) or declining firms (Lalinsky & Pál, 2021). Fourth, productivity has dwindled during the COVID pandemic, mainly because the usual selection effects, i.e., the forces of creative destruction that reallocate resources and market share towards ‘fitter’ better-performing firms, could not operate as usual amid circumstances of severe economic disruption and strong government life-support interventions. Productivity-enhancing selection effects require that resources such as finance are allocated towards the promising innovative startups that need them the most.

While firms that have experience and capabilities in dealing with public institutions appear to be more likely to benefit from public COVID support (Lalinsky & Pál, 2021), nevertheless there are concerns that some vulnerable firms might be neglected and facing extreme hardships. Furthermore, while government support schemes may prop up the liquidity of vulnerable firms temporarily, thereby reducing failure rates, there may be a “ticking time-bomb” effect whereby failure rates will shoot upwards once the support schemes expire (Gourinchas et al., 2021). Therefore, it would be valuable to learn how vulnerable firms are doing in terms of variables such as internal finance and investment plans.

In an attempt to help fill this gap in our knowledge regarding investment activity by vulnerable firms in EU member states, we contribute with new evidence regarding how vulnerable firms fare during the COVID crisis. Specifically, we focus on self-reported predictions about investment levels and investment-related framework conditions, i.e., predictions made by firms in a 2020 survey, and compare these predictions regarding investment levels with the responses to the same survey questions in previous years, to investigate whether the COVID shock has led to an unusual drop in investment plans by potentially vulnerable groups of firms. We focus on four groups of potentially vulnerable firms: high-growth enterprises (HGEs), young and small firms, R&D investors and non-subsidiary firms. We acknowledge also the strong sectoral driver of the COVID impact and we control for sectors using sector dummies, as well as sector × year fixed effects. A more detailed analysis of sectoral differences seems beyond the scope of the current paper, but we present in Appendix OSM-4 the cross-sectoral sales drop and investment behavior after the COVID impact.

Another issue motivating our research is that, in times of crisis, firms tend to change the nature of their investments: *how much* they invest, as well as *how* they invest (*i.e*., what are their investment priorities). In general, the COVID shock has brought about a dramatic drop in economic activity, which has led to heightened uncertainty (Altig et al., 2020), and which therefore can be expected to make firms and investors more risk-averse as a response to the heightened uncertainty, leading firms to shy away from risky long-term innovative projects and to focus on lower-risk investment projects such as replacing and boosting production capacity of existing goods and services. Furthermore, firms vary in terms of vulnerability to investment barriers (Alves et al., 2019), and some firms’ investment behaviors are more responsive to negative shocks than others. Garicano and Steinwender (2016) show how the Great Recession appears to have caused Spanish firms to shift their investments away from longer-term investments and towards short-term investments. Their analysis relies on applying the difference-in-difference technique for panel data. We provide new research on how (vulnerable) firms may have shifted their investment priorities after the onset of the COVID crisis.

A third motivation for our analysis relates to investment in innovation. In good times, firms are suspected of under-investing in innovation because of a range of problems such as imitation, uncertain appropriation of the benefits, and uncertainty regarding the timescale and the overall payoff of investments in innovation (Hall, 2002). In times of COVID, problems of the financing of innovation can be expected to become more severe (Roper and Turner, 2020). In fact, for the first time in a decade, EU companies decreased their overall R&D investments (Grassano et al., 2021). We therefore investigate how the COVID shock has affected the investment expectations of R&D investors.

Based on the European Investment Bank’s Group Survey on Investment and Investment Finance (EIBIS) matched with ORBIS data, this paper applies a difference-in-difference estimator to our survey data on investment expectations, to evaluate the impact of the COVID shock on investment. Crucially important to our empirical strategy is the fact that firms responded in May–August 2020 (i.e., after the onset of the COVID crisis) regarding their investment plans in the next 12 months or more, which allow us to see whether investment plans are lower for certain vulnerable groups, and also to compare these self-reported investment plans with the responses by firms to the same survey questions in previous years. We do not observe actual investment levels since the onset of the COVID shock, but we have information on firms’ expectations regarding investment in several survey waves. Of course, we have one post-COVID observation per firm regarding their expectations regarding investment activity, and compare this to the same survey question from previous years. Essentially, we investigate whether differences in post-crisis investment expectations are driven by firm-level differences, focusing in particular on whether the investment expectations of ‘vulnerable’ firms (i.e., High-Growth Enterprises (HGEs), young and small firms, R&D investors, non-subsidiary firms) differ from the investment expectations of their respective non-vulnerable counterparts. Our survey questions on expectations relating to investment activity correspond to expected investment growth, and availability of internal and external finance. Moreover, results on expectations regarding the framework conditions for investment, and the nature of the expected investment (replacing, expanding capacity, and/or developing and introducing new products or services) are presented additionally in the Online Supplementary Materials (Appendix OSM-2).

A main strength of our approach results from the panel structure of our survey data that permits a difference-in-difference approach (i.e., investigating differences in the severity of the COVID shock across different groups of firms, comparing vulnerable firms to their less vulnerable counterparts). Given the recency of the COVID shock, which is still ongoing, it is challenging to get information on post-COVID investment. Nevertheless, we exploit survey responses on self-reported forward-looking investment plans, comparing responses to these survey questions with responses to identical questions that were asked in previous years that help to establish a pre-crisis benchmark for making comparisons.

Our analysis is exploratory in nature and yields a number of interesting associations regarding sudden changes in investment expectations that may be suggestive of causal effects. We observe that R&D investors are more likely to expect to decrease their investment in the wake of the COVID shock. This can contribute to widening the gap between the EU and US. High Growth Enterprises (HGEs) expect to be less likely to increase their investment, and also have pessimistic expectations about the availability of external finance. For their part, R&D investors are pessimistic about the availability of both internal and external finance. These findings suggest that there could be a role for policy to support these firms in terms of the financing of investment (whether it be internal or external finance) or potentially to support their investment plans.

The paper unfolds as follows. Section 2 presents the data, and Sect. 3 presents our empirical methodology. Section 4 presents our regression results, which are complemented by event study graphs in Sect. 5. Section 6 concludes.

## Data

We draw on a panel database that is obtained by merging together the European Investment Bank Investment Survey (EIBIS) with the ORBIS dataset maintained by Bureau van Dijk (Coad et al., 2021). Survey data can be a valuable source of information on the investment activities of firms (Alves et al., 2019; Balduzzi et al., 2020).^1^ In our case, EIBIS contains qualitative and quantitative information on the investment activities by non-financial corporates, both SMEs (5–250 employees) as well as larger corporates (250 + employees). EIBIS also collects information on their financing requirements and the difficulties that they face. EIBIS applies stratified sampling with a goal of being representative across all countries (all 27 Member States of the EU, and also the UK), within countries, within four firm size classes (i.e., micro, small, medium and large) and within four sector groupings (i.e., manufacturing, construction, services, and infrastructure). Our analysis focuses exclusively on the 27 EU Member States. EIBIS is carried out via computer-assisted telephone interviews (CATI) in the local language. The interviewed firms are all drawn from Bureau van Dijk’s ORBIS database, which enables the linking of EIBIS survey answers to firms’ financial variables and other administrative information, while maintaining the anonymity of firms’ information. Methodological details on EIBIS are available from IPSOS.^2^ Brutscher et al. (2020) show that EIBIS is a reliable data source with no systematic sampling bias.

EIBIS contains information on around 12,500 firms in each annual survey wave from 2016 to 2020. EIBIS contains rich information on investment types in pre-COVID years (i.e. 2016–2019), such as investment in fixed assets, tangible assets, digital technologies, R&D, investment in improving energy efficiency, investment in reducing CO_2_ emissions, and investment in expected future employment. However, for many of these investment variables, we have no observation for the post-COVID period. For example, in the EIBIS 2020 wave (i.e., the “COVID wave”) the question on realized investment refers to last financial year, which is 2019. This backward-looking question is not useful for our difference-in-difference setup, because even if the question is asked to firms after the onset of COVID, it relates to information from the period before the onset of COVID. Therefore we focus only on forward-looking investment questions, which correspond to investment *expectations* rather than actual amounts invested. Regarding the 2020 survey wave, we should note that the survey is conducted between May and August, and the sampling period overlaps across countries. Therefore, it is not the case that some countries are systematically surveyed either earlier or later in the development of the COVID crisis.

The main variables used in our analysis can be subdivided into dependent variables (forward-looking responses on expected investment activity, financing conditions and business environment) and indicators for vulnerable firms (HGEs, young and small firms, R&D investors, and also subsidiary firms as a case of non-vulnerable firms) and are presented in detail in Sect. 3.3.

Some summary statistics on our groups of vulnerable firms are shown in Table 1. About 9% of firms in our EIBIS 2020 wave are HGEs, which is slightly lower than in previous years (around 12% in 2019 and 2018, see Coad et al., 2022a). About 20% of firms in our sample are R&D investors, and around 25% are subsidiaries. About 10% of firms are in our “young × small” category. Appendix OSM-3 presents summary statistics for the investment expectations of HGEs in 2019 and 2020, while Appendix OSM-5 presents summary statistics according to country and sector, as well as summary statistics for the investment variables.

**Table 1:** Summary statistics on vulnerable firms. Source: EIBIS survey, 2020 wave

Variable	Obs	Mean	Std. dev	Min	Max
HGE	11,957	0.094	0.291	0	1
R&D investors	12,572	0.205	0.404	0	1
Subsidiaries	12,571	0.254	0.435	0	1
Young × small	12,572	0.102	0.303	0	1

Please see Table 2 for variable definitions

## Methodology

### Introduction

A naive approach to estimating the Average Treatment Effect on the Treated (ATT) regarding how the COVID shock affected vulnerable (“V”) vs non-vulnerable (“NV”) groups of firms would be to compare the means of outcome variables ${Y}_{t}$ for the post-COVID survey wave *t*:

$$ATT= {Y}_{t}^{V}-{Y}_{t}^{NV}$$

The problem with this approach is that, while we have a control group of non-vulnerable firms, nevertheless we have not taken into account pre-existing differences between these two groups that might confound the interpretation of different outcomes in the post-COVID survey wave, as well as common time trends $T$.

Difference-in-difference (DiD) analysis is a quasi-experimental identification strategy for estimating causal effects in panel data that is long-established and widely used among applied econometricians (Cunningham, 2021; Roth et al., 2022). In our context, all of our firms will be “treated” at the same time, because the COVID shock struck all firms in the same survey year.^3^ A DiD approach allows us to remove (through differencing) these two confounding influences of time-invariant unobserved heterogeneity and common time trends:

$$ATT= \left({Y}_{t}^{V}-{Y}_{t-1}^{V}\right)-\left({Y}_{t}^{NV}-{Y}_{t-1}^{NV}\right)$$

In the equation above, the differencing that takes place within rounded brackets allows us to cancel out the time-invariant unobserved heterogeneity. Then, subtracting one rounded bracket from the other allows us to cancel out the effect of the common time trend.

However, an additional problem could arise in the form of time-*varying* unobserved heterogeneity, which would affect the outcomes at time t and cannot be removed by differencing. We assume that any heterogeneity between NV and V is time-invariant and not time-varying, which is known as the “parallel trends assumption” and is fundamentally untestable (Cunningham, 2021). If this assumption is not verified, then our estimates cannot be interpreted as causal effects. While untestable, nevertheless the plausibility of the parallel trends assumption can be investigated via event study graphs, that can be useful tools to explore whether the two groups had comparable dynamics in the pre-treatment period (Cunningham, 2021). We therefore present such event study graphs alongside fixed effects (“within”) panel regressions using standard errors clustered at the firm level.

### Empirical setup

**Fig. 1:**
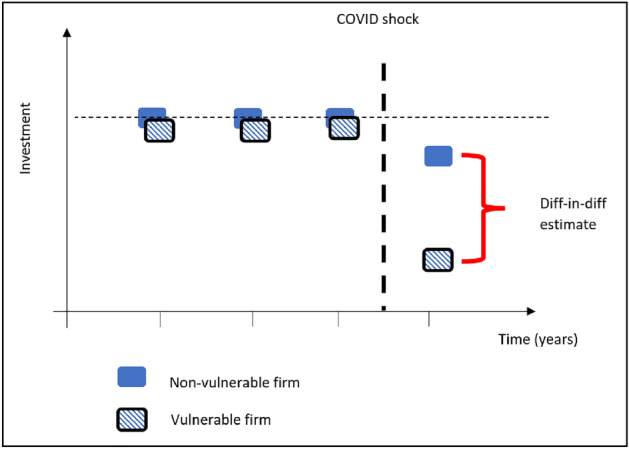
Our difference-in-difference approach. Source: our elaboration

Figure 1 gives an initial intuition behind our difference-in-difference approach. We observe pre-COVID trends for all firms (both non-vulnerable and vulnerable firms). Investment levels of these firms pre-COVID may evolve according to similar trends, or at different levels but with parallel trends, or perhaps even with diverging trends. Then, we have one observation after the COVID shock, which we use to see how different types of firms were differentially affected by the COVID shock with respect to their pre-COVID trends. The COVID shock is considered to be an exogenous event (Garicano & Steinwender, 2016). In Fig. 1, both firms had similar pre-COVID trends, but the vulnerable firm had a stronger reaction to the COVID shock than the non-vulnerable firm. We therefore look for *differences* in post-shock outcomes for *different* types of firms (*i.e*., a *difference-in-difference* approach).

Similar to Garicano & Steinwender (2016),^4^ our difference-in-difference estimator is:

1 $$\begin{aligned}{Inv}_{ict} & ={\beta }_{0}+{\beta }_{1}{crisis}_{t}\times {firm\_type}_{c}+{\beta }_{2}{crisis}_{t}+ {\beta }_{3}{firm\_type}_{c} \\ &\quad +{\beta }_{4}{CONTROLS}_{ict}+{\varepsilon }_{ict}\end{aligned}$$

For firm *i* in year *t*, where each firm is put into a binary category $c=\left\{vulnerable, non-vulnerable\right\}$ to investigate whether some types of firms (*i.e*., HGEs, young and small firms, R&D investors, non-subsidiary firms) have different outcomes when compared to their non-vulnerable counterparts (HGEs vs non-HGEs, R&D investors vs non-R&D investors, etc.). The dependent variable ${Inv}_{ict}$ corresponds to self-reported survey responses regarding investment. The dependent variable is not continuous but binary, therefore our regressions are linear probability models (LPMs, Angrist & Pischke, 2008).^5^ There is a dummy variable ${crisis}_{t}$ that is equal to 1 in the post-crisis years after the COVID shock, where ${firm\_type}_{c}$ corresponds to the type of firm (according to its vulnerability). We note that the type of firm is defined based on pre-COVID data.

Of primary interest is the coefficient ${\beta }_{1}$ that corresponds to the difference-in-difference estimate: whether the investment of vulnerable firms has reacted more strongly than the investment of non-vulnerable firms, specifically in the post-COVID survey.

In our case, due to data limitations, we have only one post-COVID observation, that is a subjectively-reported question on investment plans. This same variable is available in the survey waves corresponding to pre-COVID and post-COVID years.

### Variables

The following subsections present our dependent variables and also our categories of vulnerable firms. These variables are summarized in Table 2.

**Table 2:** Description of the main variables. Source: EIBIS survey

Dependent variables (binary variables)	
Summary	Wording of the survey question
Expected change in investment (positive dummy; negative dummy)	For the current financial year, do you expect your total investment spend to be: A. More than last year; B. Around the same amount as last year; C. Less than last year; D. No investment planned. Coded into two dummy variables corresponding to positive (vs non-positive); or negative (vs non-negative)
Availability of internal finance (improvement dummy; deterioration dummy)	Do you think that each of the following will improve, stay the same, or get worse over the next 12 months? A. Availability of internal finance within the company (e.g. internal funds like cash). Possible answers: Improve; Stay the same; Deteriorate. Coded into two dummy variables corresponding to improve (vs non-improve); or deteriorate (vs non-deteriorate)
Availability of external finance (improvement dummy; deterioration dummy)	Do you think that each of the following will improve, stay the same, or get worse over the next 12 months? B. Availability of external finance (e.g. bank financing, private or public equity). Possible answers: Improve; Stay the same; Deteriorate. Coded into two dummy variables corresponding to improve (vs non-improve); or deteriorate (vs non-deteriorate)
Industry’s business prospects* (improvement dummy; deterioration dummy)	Do you think that each of the following will improve, stay the same, or get worse over the next 12 months? C. Business prospects specific to your sector or industry. Possible answers: Improve; Stay the same; Deteriorate. Coded into two dummy variables corresponding to improve (vs non-improve); or deteriorate (vs non-deteriorate)
Overall economic climate* (improvement dummy; deterioration dummy)	Do you think that each of the following will improve, stay the same, or get worse over the next 12 months? D. Overall economic climate. Possible answers: Improve; Stay the same; Deteriorate. Coded into two dummy variables corresponding to improve (vs non-improve); or deteriorate (vs non-deteriorate)
Political and regulatory climate* (improvement dummy; deterioration dummy)	Do you think that each of the following will improve, stay the same, or get worse over the next 12 months? E. Political and regulatory climate. Possible answers: Improve; Stay the same; Deteriorate. Coded into two dummy variables corresponding to improve (vs non-improve); or deteriorate (vs non-deteriorate)
New products/processes*	Investment priority in the next three years: A. Developing or introducing new products, processes or services
Replacing capacity*	Investment priority in the next three years: B. Replacing capacity (including existing buildings, machinery, equipment and IT)
Capacity expansion*	Investment priority in the next three years: C. Capacity expansion of existing production facility
*Proxy for "vulnerable" firms (binary variables)*	
Subsidiary	If the firm is a subsidiary
R&D	If the firm has positive R&D investment
HGE	If the firm is a High Growth Enterprise. HGEs are defined in our EIBIS panel as enterprises with an average annualized employment growth of 10% or more per year over the past three years, as well as having 10 or more employees at the beginning of the growth period. Our HGE definition is similar to the standard OECD-Eurostat definition of HGEs (Eurostat-OECD, 2007)
Young × small	Interaction term of the variables young and small. ‘Young’ refers to a dummy for firms younger than 10 years old, while ‘small’ refers to a dummy variable that equals 1 if the firm has up to 49 employees

Asterisks * indicate that results for the dependent variables relating to the broader business environment and economic political climate, as well as firms’ investment priorities (new products/processes; replacing capacity; capacity expansion) are shown in Appendix OSM.2

#### Dependent variables

Our difference-in-difference approach focuses on variation in self-reported forward-looking investment variables (*i.e*., the variable ${Inv}_{ict}$ from Eq. (1) above), that in the latest EIBIS survey wave correspond to predicted investment after the onset of the COVID shock:

- For the current financial year, do you expect your total investment spend to be… A. More than last year; B. Around the same amount as last year; C. Less than last year; D. No investment planned.
- Do you think that each of the following will improve, stay the same, or get worse over the next 12 months? A. Availability of internal finance within the company (*e.g*., internal funds like cash); B. Availability of external finance (*e.g*., bank financing, private or public equity); C. Business prospects specific to your sector or industry; D. Overall economic climate; E. Political and regulatory climate. [For each of these 5 questions, answers are: Improve; Stay the same; Deteriorate]
- And looking ahead to the next three years, which of the following is your investment priority? A. Developing or introducing new products, processes or services; B. Replacing capacity (including existing buildings, machinery, equipment and IT); C. Capacity expansion for existing products/services; D. Or do you have no investment planned?

Our analysis relating to business prospects and the economic and political climate (second-last bullet point) as well as the analysis relating to investment priorities, referred to in the last bullet point, are not investigated in the main text, but appear in Appendix OSM-2.

#### Groups of vulnerable firms

A central dimension of our analysis is the distinction between vulnerable and non-vulnerable firms. Some firms may be more vulnerable than others, in particular with regards to their investment needs and outcomes in times of crisis. Policymakers may be interested in knowing whether, and to what extent, certain types of firms (that are of considerable policy interest) may be more vulnerable in times of crisis, for example SME policy and entrepreneurship policy which focus on providing support to firms perceived as vulnerable (e.g. young small innovative high-growth firms). High-potential firms (such as HGEs and R&D investors) that risk being disproportionately affected by the COVID shock may also be categorized as vulnerable. The variables that we consider to correspond to vulnerable firms, and a short justification for their characterization as being potentially “vulnerable”, are as follows:

- HGE vs non-HGE. High-growth enterprises (HGEs) make a disproportionate contribution to economic growth and job creation, and as a result they have attracted considerable attention from policy-makers (Benedetti Fasil et al., 2021; Flachenecker et al., 2020). In good times, HGEs may be vulnerable because of the dangers of rapid growth (Coad et al., 2020), or the high costs of growth (Rostamkalaei & Freel, 2016), or the difficulties of overcoming growth barriers such as requirements for skilled labor. In times of crisis, HGEs may be especially vulnerable because of a tightening of credit and a decrease in confidence.
- Young vs old. Young firms are uniquely vulnerable, given their lack of reputation, absence of routines, and that co-workers lack job tenure experience of working together (Coad, 2018). The liabilities of young age seem to be particularly severe in the first 5–7 years of life (Coad, 2018). Nevertheless, young firms play a unique role in terms of rapid growth and job creation (Cincera & Veugelers, 2013; Haltiwanger et al., 2013; Pellegrino & Piva, 2020). Young firms are particularly vulnerable if they are also small, because in this case they lack the resources that could help them overcome problems related to lack of reputation and track-record.
- Small vs large. Small firms, in particular, have been singled out as being vulnerable to obstacles to investment (Alves et al., 2019), especially in times of COVID (Balduzzi et al., 2021). Small firms are also more vulnerable to insolvency, even in pre-COVID times and of course also in post-COVID times (Lalinsky & Pál, 2021). Small firms are measured here in terms of a dummy variable that equals 1 if the firm has up to 49 employees. While old small firms may have a reputation and experience to help them overcome challenges linked to their small size, young small firms can be expected to be particularly vulnerable. We therefore investigate the role of age and size by applying an interaction term that focuses specifically on firms that are both young and small.
- R&D investors vs non-investors. R&D investment contributes to innovation and economic growth, and as a result policymakers seek to encourage R&D investment (Moncada-Paternò-Castello, 2022; Zhang & Mohnen, 2022). Nevertheless, R&D investors are vulnerable in many dimensions: sensitive to uncertainty and long payback times in their innovation investment projects, having low levels of collateral and vulnerable to information asymmetries when seeking finance. Marques Santos et al. (2021) observe that the impact of the COVID crisis on firms’ turnover growth was smaller for innovative than for non-innovative firms.
- Subsidiaries vs non-subsidiary firms. Subsidiaries are assumed to have relatively low levels of vulnerability, in the sense that they are part of a larger business group and, in times of crisis, they may be able to access the financial “deep pockets” of the parent company (Khanna & Yafeh, 2007).

Table 2 summarizes the information on our variables. Altogether, we have 4 indicators for whether a firm is vulnerable. Given the exploratory nature of our research, which features a large number of dependent variables relating to investment, and also the large number of indicators for vulnerable firms, we have opted to not develop a set of specific hypotheses (Helfat, 2007). Instead, our broad research question focuses on whether the investment plans of these different types of vulnerable firms are hit disproportionately hard after the onset of the COVID crisis.

We also seek to include control variables in our regressions. On the one hand, to facilitate comparisons, we seek to have the same set of control variables in each regression, to facilitate comparisons across groups of vulnerable firms. On the other hand, this is not always possible, because we cannot include as controls those variables that are sometimes taken as proxies for vulnerable firms themselves (e.g. firm size, firm age). Moreover, recall that time-invariant firm-specific variables will be absorbed into the firm fixed effects that are included in our regressions. Therefore, we use a standardized set of controls to facilitate comparisons across regressions (in line with Cirera et al., 2021). In some cases, potentially interesting control variables are not included, because they are affected by many missing values and would hence reduce the sample size (*e.g*., investments in ICT and digitalization). Therefore, our set of control variables refers to country, year, industrial sector and firm-specific dummies (*i.e*., firm fixed effects), as well as country × year and sector × year fixed effects, and a constant term.^6^

Our application of the differences-in-differences (DiD) estimator seeks to follow recommendations for best-practice (*e.g*., Cunningham, 2021 and Huntington-Klein, 2021). One potentially tricky aspect of difference-in-differences regression arises when treatments affect participants at different times (Goodman-Bacon, 2021), although in our context the outbreak of the COVID crisis hits all firms at the same time (Garicano & Steinwender, 2016). Another potentially tricky aspect of difference-in-differences regression relates to the parallel trends assumption (*i.e*., the possibility that differences between groups of firms at time *t* are simply extrapolations of previously diverging trends in the years leading up to *t*). The parallel trends assumption is fundamentally untestable (Cunningham, 2021), although we investigate it by showing the investment trends for vulnerable and non-vulnerable firms in the years before the crisis in graphical form. To this end, Appendix OSM-1 contains 15 × 4 = 60 graphs that the curious reader may peruse. Given the challenges surrounding the parallel trends assumption (which is necessary if we are to ascribe causal interpretations to our results), instead we advise the reader to interpret our results conservatively as associations rather than causal effects.

#### Expectations as a proxy for actual investment

We investigate whether the COVID shock has disproportionately affected vulnerable firms by using expectations surrounding investment as a proxy for actual investment (Balduzzi et al., 2020). This empirical approach seems necessary, because the COVID shock appeared so recently that data on actual post-COVID firm-level investment is not available to us yet. We cannot check whether investment expectations in the 2020 EIBIS wave closely correspond to actual investment, however, the next best thing could be to check whether the same question in the previous survey wave (EIBIS 2019) corresponds to actual investment as reported in EIBIS 2020.

The exact wording of the survey question on expected investment in 2019 is in Table 2. The actual growth rate of investment is calculated using log-differences as:

$${gr\_inv}_{2019-2020}={log\left(total\_investment\right)}_{2020}-{log\left(total\_investment\right)}_{2019}$$

**Fig. 2:**
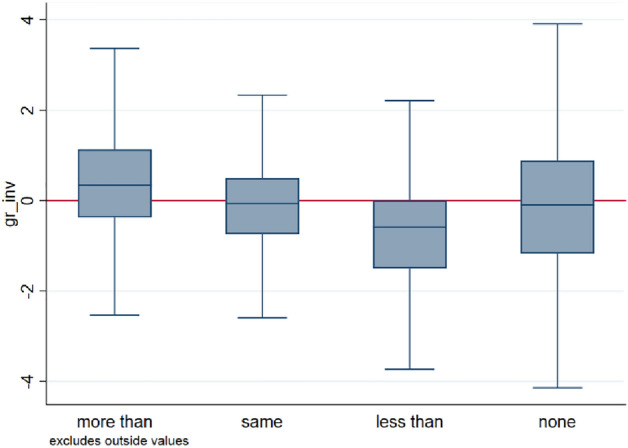
Expected investment (reported in EIBIS 2019) versus actual growth of investment over the period 2019–2020 (using information reported in EIBIS 2020). Source: EIBIS survey, our analysis. Notes: Horizontal red line corresponds to growth rates of 0%

Figure 2 shows that expectations surrounding investment are a meaningful approximation of actual investment, especially considering that information on actual investment is simply not available to us in the latest EIBIS survey wave. Figure 2 shows that firms that expect to invest more have a higher average growth of investment than firms that expect to invest the same amount, who in turn have a higher average growth of investment than firms that expect to invest less than in the previous year. For the category “less than”, about 75% of firms have non-positive growth rates of investment. The category of “none” (*i.e*., those firms reporting that they have no investment planned) seems a bit of an exception to the pattern for the three other categories. Overall, we consider that investment expectations do not perfectly predict actual investment, but they are certainly much better than random noise, and we take them as a meaningful proxy variable for actual future investment activity. Investment expectations may correspond better to the foreseeable or strategic or longer-term components of investment expenditure, as opposed to short-term ‘surprise’ investment obligations, although of course we have no way of testing this conjecture.

## Regression results

Equation (1) is estimated using fixed effects (also known as “within”) panel regressions that remove the time-invariant unobserved heterogeneity that could be affecting firm investment behavior. Country × year and sector × year fixed effects control for the possibility that countries and sectors may have idiosyncratic dynamics that could potentially obscure the firm-level dynamics that we wish to focus on. Given that we have many alternative dependent variables, and many alternative indicators for what is a “vulnerable” group of firms, this gives us a rich set of regression results. Tables 3 and 4 summarize these fixed effects regression results tables by reporting the DiD coefficient (*i.e*., the coefficient of ${\beta }_{1}$ from Eq. (1)).^7^

**Table 3:** Estimates of the DiD coefficient of ${\beta }_{1}$ obtained from FE (i.e. within) regressions of Eq. (1). Source: EIBIS survey, our analysis

	HGE	Subsidiary	R&D	Young × small
Expected change in investment: negative	− 0.226	**3.940**	**4.209**	− 3.734
	(− 0.08)	(2.11)	(2.07)	(− 1.40)
Expected change in investment: positive	**− 5.541**	− 1.996	− 1.617	− 1.490
	(− 2.03)	(− 1.13)	(− 0.86)	(− 0.61)

Coefficients significant at the 5% level appear in bold. Controls include country × year and sector × year fixed effects. This table summarizes results from 2 × 4 = 8 different regressions (i.e., 2 alternative dependent variables and 4 alternative proxies for “vulnerable” firms). The table shows coefficients as well as t-statistics that are obtained after clustering the standard errors at the firm level. Control variables, constant term, and model fit statistics for the regressions are not shown here for conciseness. Results available from the authors upon request

**Table 4:** Estimates of the DiD coefficient of ${\beta }_{1}$ obtained from FE (i.e. within) regressions of Eq. (1). Source: EIBIS survey, our analysis

	HGE	Subsidiary	R&D	Young × Small
Availability of internal finance: negative	− 2.352	− 1.197	1.733	2.947
	(− 1.02)	(0.77)	(1.03)	(1.30)
Availability of internal finance: positive	− 3.270	0.087	**− 4.847**	**− 5.575**
	(− 1.30)	(0.06)	(− 3.02)	(− 2.47)
Availability of external finance: negative	**6.328**	− 0.265	**4.428**	− 3.031
	(2.62)	(− 0.18)	(2.60)	(− 1.26)
Availability of external finance: positive	**− 5.605**	− 1.850	− 0.677	− 2.411
	(− 2.13)	(− 1.14)	(− 0.37)	(− 0.96)

Coefficients significant at the 5% level appear in bold. Controls include country × year and sector × year fixed effects. This table summarizes results from 4 × 4 = 16 different regressions (i.e. 4 alternative dependent variables and 4 alternative proxies for “vulnerable” firms). The table shows coefficients as well as t-statistics that are obtained after clustering the standard errors at the firm level. Control variables, constant term, and model fit statistics for the regressions are not shown here for conciseness. Results available from the authors upon request

Our regression results tables (Tables 3 and 4) show that, most of the time, the DiD coefficients are not statistically significant.

### Expectations regarding total investment

Table 3 shows the regression results for firms’ investment expectations. This issue is of interest to policymakers who are concerned about whether vulnerable firms are expecting to cut investment more than non-vulnerable firms.

HGEs could be a cause for concern, given that they are significantly less likely to report a positive expected change in total investment. A closer inspection (presented in our subsequent graphical analysis) shows that while in pre-COVID times HGEs usually expect to invest more than non-HGEs, and that post-COVID HGEs still have larger investment expectations than non-HGEs, nevertheless HGEs have been disproportionately badly affected by the COVID shock in the sense that the change in investment activity of HGEs has dropped faster than for non-HGEs. R&D investors may be a cause for concern too, given that they are more likely to report a negative expected change in total investment.

Subsidiary firms, which can be considered to be a case of non-vulnerable firms, have seen their investment plans affected by COVID in that they are more likely to report a negative expected change in total investment, and less likely to report a positive expected change in total investment.

Summarizing for vulnerable firms, Table 3 suggests that it is potentially concerning that R&D investing firms and HGEs are somewhat pessimistic about their investment plans post-COVID. Young and small firms, however, have not had their overall investment plans significantly affected by the COVID shock (as far as we can tell from our regressions).

### Expectations regarding investment issues

Table 4 shows that HGEs have been negatively affected by the COVID shock. HGEs are significantly more likely to be pessimistic about the availability of external finance, and significantly less likely to be optimistic.

With regards to young and small firms, the only coefficient that is statistically significant indicates that young and small firms are significantly less likely to be positive about the availability of internal finance.

Regarding R&D investors, the results in Table 4 are somewhat concerning. R&D investors are less likely to report positive expectations regarding the availability of internal finance. Moreover, they are more likely to report negative expectations regarding the availability of external finance.

### Summarizing the regression results

Our regression results can also be discussed by focusing on one category of vulnerable firm at a time.

HGEs report that they are less likely to expect a positive change in investment, following on from the COVID shock. This could be interpreted as a consequence of the vulnerable status of HGEs in the face of negative economic shocks. HGEs are also pessimistic about the availability of external finance. Hence, a possible deterioration of their access to external finance could be an area for policy makers to keep in mind.

Young and small firms did not seem to have had their overall investment plans affected by the COVID shock in a statistically significant way. Nevertheless, they are less likely to be optimistic about the availability of internal finance.

Subsidiary firms are a group of firms that are selected here as being less vulnerable to the COVID shock, because they may benefit from the support and experience of the business group, as well as potentially having access to the group’s ‘deep pockets’ of financial resources. Subsidiary firms, interestingly, are more likely to report a decrease in expected investment. This could be part of a conservative group-level strategy to brace for lean times. Subsidiary firms could be engaging in a coordinated group-level reduction in investment that is not necessarily caused by any detectable lack of access to (internal or external) finance.

R&D investing firms are more likely to expect to decrease their investment levels, which could be a cause for concern. R&D investors are also less likely to report improvements in availability of internal finance, and more likely to be pessimistic about the availability of external finance.

Overall, therefore, the COVID shock has been followed by a significant drop in the expected investment of R&D investors. R&D investors seem to be concerned about the availability of external finance, and are also less optimistic about the availability of internal finance. The COVID shock is also associated with HGEs being less likely to expect to increase their investment activity, which could be linked to their dismal expectations about the availability of external finance. Young and small firms are observed to be less optimistic about the availability of external finance.

These regression results are useful for providing estimates of the DiD coefficient (*i.e*., the coefficient of ${\beta }_{1}$ from Eq. (1)) and checking their statistical significance and overall model fit, while controlling for the potentially confounding influence of control variables. However, these regressions can be enriched with an analysis of event study plots that provide insights on pre-crisis trends (*i.e*., the parallel trends assumption).

## Event study graphs

**Fig. 3:**
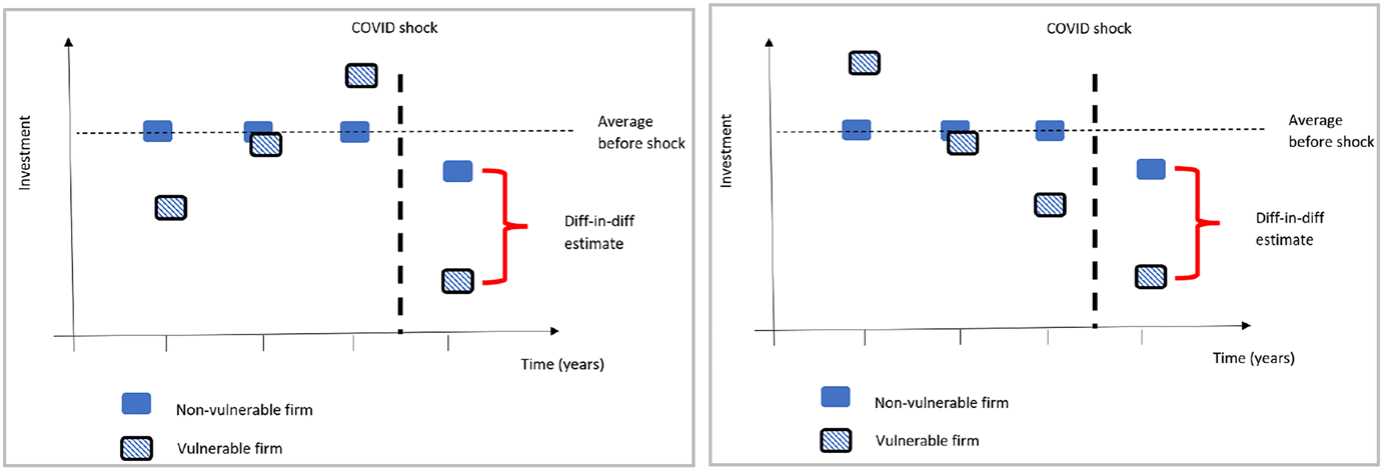
scenarios of the difference-in-difference approach. While our difference-in-difference regressions cannot clearly distinguish between these two cases, nevertheless they are distinguishable in event study graphs. Source: our elaboration

In this section, event study graphs (Cunningham, 2021) are shown to highlight the differences between vulnerable groups (e.g., HGEs, R&D investors) and their complement (i.e., their non- vulnerable counterparts), with respect to responses to the COVID shock. We are not only interested in whether vulnerable groups are different in 2020, but whether they were also different in 2019 and earlier, and event study graphs are a useful tool in this respect. Event study graphs can therefore be a useful complement to the regression results in Tables 3 and 4, because they can shed light on the trends leading up to the shock. Figure 1 corresponds to the case where both non-vulnerable and vulnerable firms stay at their average values in the periods leading up to the COVID shock, hence the “parallel trends” assumption is supported (Cunningham, 2021) and differences between non-vulnerable and vulnerable firms cannot be ascribed to pre-existing differences in trends. However, the cases presented below (Fig. 3) show very different scenarios, where the COVID shock either corresponds to a sudden catastrophic shock for vulnerable firms (Fig. 3, left) or a smooth continuation of previous trends (Fig. 3, right). In the former case, policymakers might be concerned to see that vulnerable firms have been particularly badly affected by the shock, whereas in the latter case, policymakers may wonder whether the shock had any particular effect at all (considering the previous trends). Note that our difference-in-difference regressions cannot clearly distinguish between these three cases (Figs. 1, 3 left, right), because in all three cases the average values for pre-crisis years are the same.

This section therefore presents event-study graphs, that are plotted after taking into account a rudimentary set of control variables (i.e., country and sector fixed effects).^8^ To be precise, the datapoints used in the event-study graphs are obtained from year-wise cross-sectional OLS regressions, hence these estimates are not directly comparable to our regression coefficients in Tables 3 and 4, although they focus on the same phenomena using the same data and related techniques. These graphs should therefore be seen as providing complementary evidence from a different angle. While regression results are evaluated with regards to whether the coefficients are statistically significant, the results shown in these event-study graphs are evaluated with regards to whether the post-COVID datapoint is merely a continuation of previous trends, or whether it is a marked disruption from previous trends. Error bars (dashed lines) on the graphs correspond to robust standard errors around the main coefficients, to indicate whether the coefficients are statistically significantly different from zero at the α = 5% level.

While space limits prevent us from showing all graphs in the print version (see instead the online Appendix OSM-1), a hand-selected set of plots will be shown here while discussing our results. Clearly, there is a risk of cherry-picking results if we present only the better-looking graphs. We respond to this valid concern in four ways. First, the reader is encouraged to inspect all the graphs in the online Appendix OSM-1 and reach their own conclusions. Second, we try to take a broad view of our results, prioritizing results that emerge to be significant and coherent across alternative indicators. Third, we also comment on non-significant results that are surprising and that differ from our prior expectations. Fourth, we remind the reader to be cautious when interpreting our results, that causal interpretations of our results are not necessarily warranted, and that policy interventions should not be based on our evidence alone but by drawing on a broader evidence base.

### Expectations regarding total investment

**Fig. 4:**
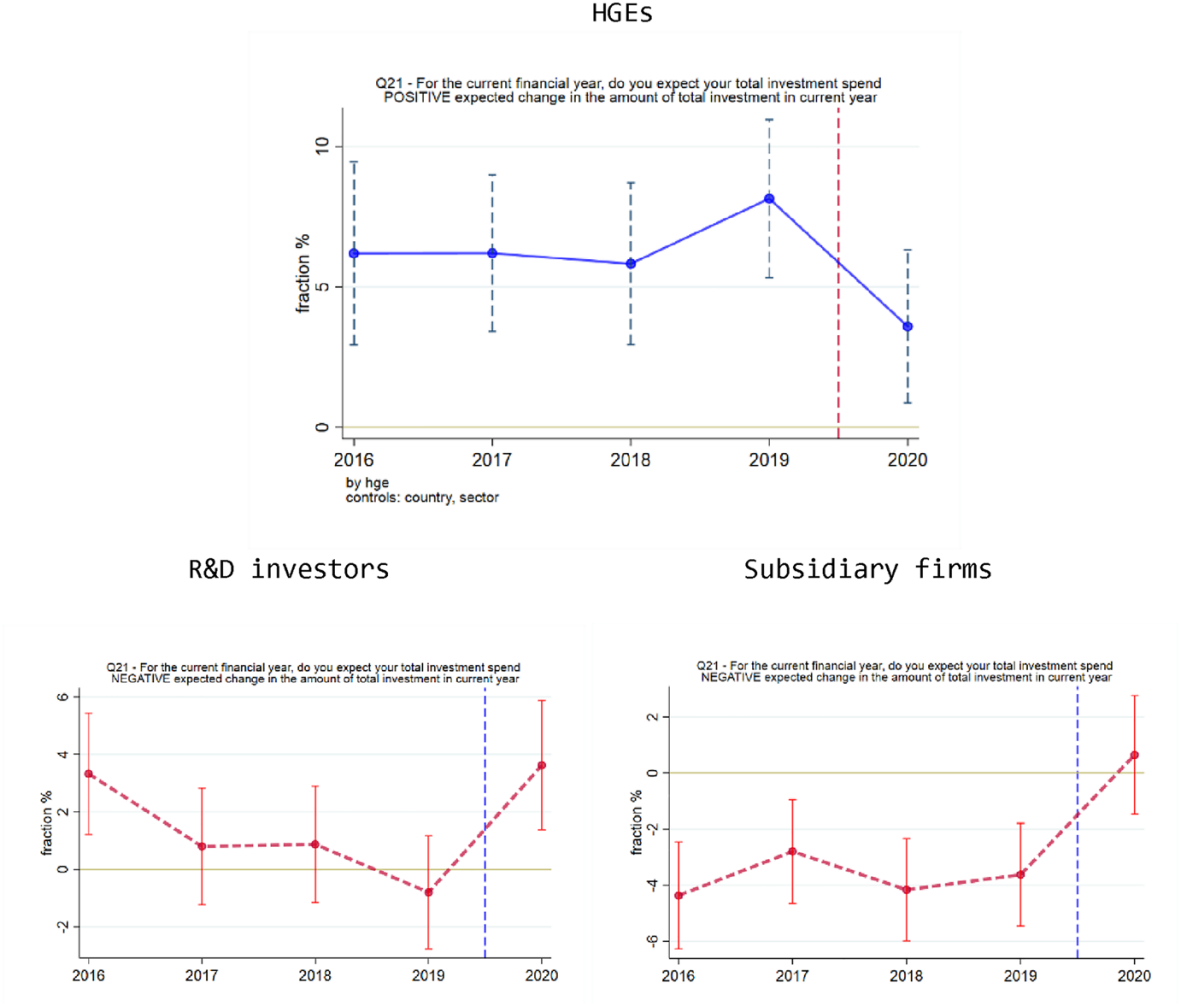
Responses regarding changes in expected total investment. Line colors differ (blue or red) because the questions differ (positive or negative expected change). Top: HGEs, positive expected change. Bottom Left: R&D investors, negative expected change. Bottom Right: Subsidiary firms, negative expected change. Source: EIBIS survey, our analysis. NOTES: Datapoints obtained from regressions that include control variables (sector and country dummies), using robust standard errors

We begin our analysis of the graphs by focusing on expectations regarding total investment. To illustrate how to interpret the graphs, we start by walking through the case of Fig. 4 (top). The horizontal axis refers to the year (2016–2020) in which the firm reported its expectations regarding total investment. The vertical axis, in percentage points, refers to the conditional difference between HGEs and non-HGEs (or, more generally, between X and non-X in the case of the X-dummy for the vulnerable group X). For example, if 25% of HGEs expect to invest more in 2020, and 19% of non-HGEs expect to invest more in 2020 (as shown in Appendix Table OSM-3), this corresponds to an unconditional difference of 6%, hence the datapoint would align with the value 6% on the horizontal axis. Positive values presented in the chart (Fig. 4, top) means that higher share of HGEs compared to non-HGEs expect to invest more. The conditional difference is not the same as the unconditional difference, because it applies control variables (here: country dummies and sector dummies) to adjust for differences between HGEs and non-HGEs in the context of an OLS regression, making the two more comparable. As it happens, Fig. 4 (top) shows that the datapoint for 2020 does not clearly emerge from extrapolating from a smooth trend in previous years (2019, 2018, and before).

The graphical results are shown in Fig. 4 and are generally in line with the regression results shown earlier in Tables 3 and 4. In most cases, the results do not show any clear changes due to COVID. In a few cases, though, the COVID shock seems to have had a distinct effect, and in the following we focus on presenting those cases where we document a relatively clear-cut COVID effect.

HGEs appear to become pessimistic regarding their expected investment in the current financial year, because they are less likely to report a positive change in investment. This finding of lower chances of expected investment growth for HGEs was observed earlier in Table 3. In the pre-COVID years, the coefficients in Fig. 4 (top) show that HGEs are about 6–8 percentage points more likely than non-HGEs to report a positive expected change in total investment, while for the year 2020 this number drops to around 4 percentage points. Hence, the HGE premium remains positive (indicating that HGEs continue to be more likely than non-HGEs to report a positive expected change in total investment), although the decrease in magnitude of the HGE premium suggests that they have been disproportionately affected by the COVID shock (compared to non-HGEs) in terms of investment expectations.

Taking a different angle on the same phenomenon of investment expectations, we focus on expectations regarding negative changes in investment. R&D investors and subsidiary firms are more likely to expect a negative change in investment. While R&D investors are usually considered to be vulnerable to financial constraints, subsidiaries may be withholding their investment as a ‘wait-and-see’ tactic (Table 4). These sudden increases in negative expectations for investment for these two groups (subsidiaries, R&D investors) were all seen to be statistically significant in Table 3 also. Hence, the event study graphs in Fig. 4 are broadly in line with the findings from our earlier regressions.

### Expectations regarding internal and external finance

Next, we investigate how the COVID shock has affected expectations regarding the availability of internal finance within the company (*e.g*., internal funds such as cash) and external finance such as bank financing, private equity or public equity.

**Fig. 5:**
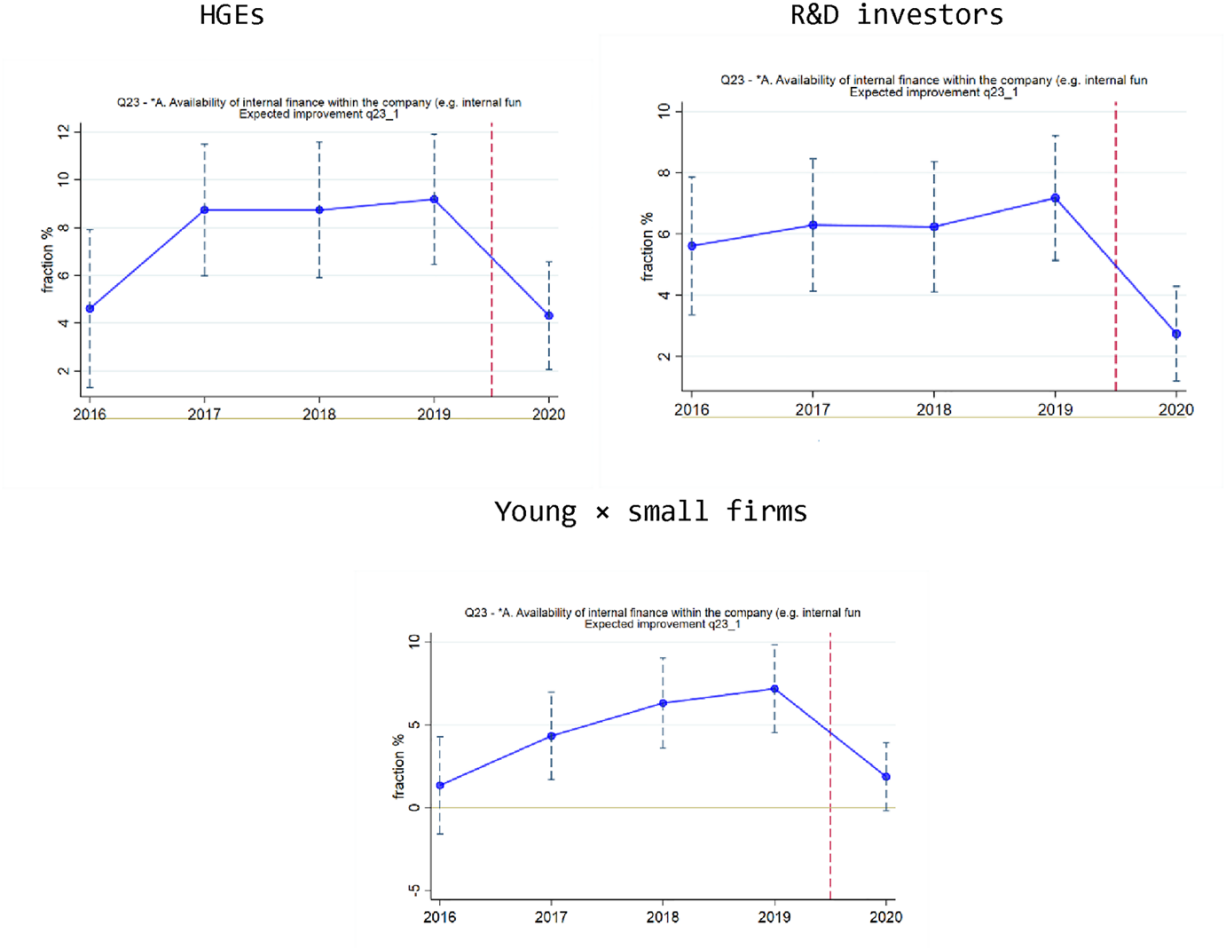
Responses regarding availability of internal finance. Top Left: HGEs, expected improvement. Top Right: R&D investors, expected improvement. Bottom: young × small firms, expected improvement. Source: EIBIS survey, our analysis. NOTES: Datapoints obtained from regressions that include control variables (sector and country dummies), using robust standard errors

Starting with the case of internal finance, the results in Fig. 5 are generally in line with the regression results in Table 4. Groups of vulnerable firms (HGEs, R&D investors, and young × small firms) are less likely to expect an improvement regarding internal finance, and the time trends seem to suggest that this decrease in optimism is a feature of the 2020 COVID survey wave. In these three cases (HGEs, R&D investors, young × small firms), the regression results in Table 4 were statistically significant for R&D investors and young × small firms, but not for HGEs. Overall, therefore, the graphs shown in Fig. 5 suggest that the situation for internal finance has deteriorated in the wake of the COVID shock.

**Fig. 6:**
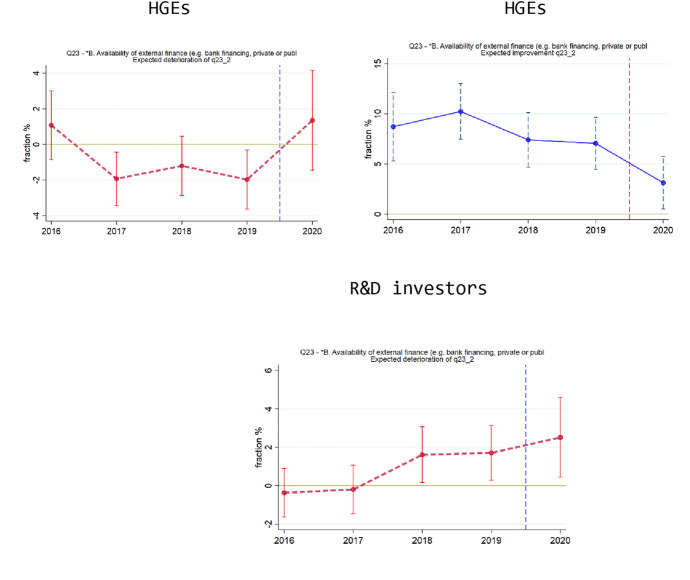
Responses regarding availability of external finance. Line colors differ (blue or red) because the questions differ (positive or negative expected change). Top Left: HGEs, expected deterioration. Top Right: HGEs, expected improvement. Bottom: R&D investors, expected deterioration. Source: EIBIS survey, our analysis. NOTES: Datapoints obtained from regressions that include control variables (sector and country dummies), using robust standard errors

Figure 6 presents graphs regarding availability of external finance, focusing mainly on the cases that are statistically significant in Table 4. The top row of Fig. 6 relates to HGEs, showing that they are more likely to expect a deterioration in the availability of external finance, and also less likely to expect an improvement. Figure 6 (bottom) shows that R&D investors are more likely to expect a deterioration in the availability of external finance.

## Conclusion

The sudden onset of the COVID shock has left European economies reeling, resulting in a sudden contraction of demand that has hit some vulnerable firms and sectors in a remarkably uneven way. As a result, there is a genuine interest from policymakers to learn about which types of firms have been left in vulnerable circumstances as a result of the crisis. While the EU and Member States have quickly set up initiatives to support vulnerable firms during the COVID crisis, nevertheless there is a need to shed light on which firms are more vulnerable.

We present new evidence on the evolution of investment plans of certain groups of firms suspected of being vulnerable (young and small firms, High-Growth Enterprises (HGEs), R&D investors and non-subsidiary firms). We are interested in seeing if these vulnerable firms’ expectations regarding investment activity have been hit especially hard by the sudden onset of the COVID crisis. To do this, we apply a difference-in-differences approach on panel data regarding forward-looking investment expectations. While vulnerable groups may generally be considered to invest more (*e.g*., HGEs or R&D investors) than non-vulnerable groups in good years, there are concerns that the COVID shock may have had an unusually severe effect on the investment plans of vulnerable firms.

Our results show that HGEs are suddenly less likely to expect a positive change in investment, while R&D investors are suddenly more likely to expect a negative change in investment. R&D investors are less likely to be optimistic about the availability of internal finance for investment purposes. HGEs and R&D investors are more likely to be pessimistic about the availability of external finance.

Overall, therefore, a particularly vulnerable group of firms seems to correspond to R&D investors, who have decreased their expectations regarding investments, and who expect problems regarding the availability of both internal and external finance. Further analysis (results presented in Appendix OSM2) shows that R&D investors are also pessimistic about their industry’s business prospects, which could be problematic given that R&D investment is procyclical and that a drop in economic confidence could lead to a decrease in R&D investment (Barlevy, 2007; Roper & Turner, 2020). In fact, EU companies have decreased their R&D investment for the first time in 10 years (Grassano et al., 2021), which could contribute to widening further the gap between the US and EU (Moncada-Paternò-Castello, 2022). Other areas of concern for policy makers could be the availability of external finance for HGEs. In this context, policy measures to alleviate liquidity concerns for vulnerable firms in times of crisis should also reflect their specific financing requirements, as for instance Benedetti-Fasil et al. (2021) point out in the case of HGEs. More generally, our results also underline the importance of policy measures as a response to economic crisis to support demand and thus stabilizing investment (expectations) across different groups of (vulnerable) firms, *e.g*., by implementing the Recovery and Resilience Facility.^9^ Understanding the needs of these types of firms is particularly relevant as actions are being considered in order to achieve a stronger and more competitive industry in Europe. Young and small firms, HGEs, and R&D-investing companies are the most dynamic segment of the corporations with highest employment and value-added growth. Despite their better performance in general, the relatively stronger negative adjustments to the shock should be alarming, and specific policy interventions focusing on investment should be implemented. In particular, risk-sharing instruments, such as guarantees and equity or quasi-equity financing are among the best fit for HGEs and R&D companies (Coad et al., 2022b). There is also considerable evidence that innovative start-up/ scale-up firms (corresponding to all categories of young and small firms, HGEs and R&D investors and non-subsidiary firms) need alternative financing solutions beyond traditional bank finance, and further development of the venture debt and venture capital markets in Europe would boost these firms, providing a helping hand during the shock and preventing negative adjustments. Such financing instruments could also be conditioned towards the new strategic targets of the Recovery and Resilience Facility, making these group of firms the frontrunners of digital and green transition.

Regarding our difference-in-difference methodology: In many cases, the parallel trends assumption was not supported: i.e., vulnerable firms may be significantly different from non-vulnerable firms in the 2020 COVID survey wave, but this can be explained by pre-COVID trends rather than being an unambiguous consequence of the COVID shock. This is a strength of our difference-in-difference panel data strategy, because other approaches would not have been able to obtain these insights. Indeed, our empirical approach combines panel regressions with complementary graphs of the dynamic evolution of investment expectations around the time of the COVID shock, to enable a deeper understanding of the phenomena.

Our study is not without limitations, which can be mentioned here. First, the groups of firms that we consider to be a priori vulnerable are young and small firms, HGEs, R&D investors and non-subsidiaries. Although these indicators for vulnerable firms yield a rich set of results, future research could extend our analysis with other possible indicators of firm-level vulnerability that are not investigated in depth here.^10^ Second, a limitation of our analysis is that we use data on the expectations of firms regarding investment activity and investment-related framework conditions. Expectations regarding investment activity may not correspond well to actual investment activity, for example if firms cannot accurately predict their future behavior or if firms suffer from the usual self-report biases. In our context, however, this simplifying assumption seems necessary because data on actual investment amounts are not available yet. Third, although we recognize the multifaced nature of the COVID shock, we do not investigate the channels through which COVID affected investment activity. Some possible channels could be changes to capacity utilization within firms, changes to demand, changes in financing conditions, changes due to increased uncertainty, and so on. Future research might be able to investigate the role of these channels in explaining how the COVID shock has affected vulnerable firms.

Overall, we have focused our discussion on a handful of statistically significant results, although we also highlight that some of our results were not statistically significant. We are aware of the dangers of cherry-picking statistically significant results. We have taken several steps to investigate whether our results are robust and reliable. Nevertheless, we hope that our results will contribute to an emerging evidence base that provides a richer understanding of how crises (such as the COVID shock) affect the investment decisions of vulnerable firms.

## Supplementary Information

Below is the link to the electronic supplementary material.

Supplementary file1 (DOCX 2023 kb)

## Footnotes

1 Our analysis focuses on the EIBIS & ORBIS merged panel, although (as it happens) the variables that we use all come from EIBIS.

2 Please see https://www.eib.org/attachments/eibis-methodology-report-2019-en.pdf (last accessed 14th March 2022).

3 The fact that, in our context, all firms are treated in the same period simplifies our econometric approach considerably (Roth et al., 2022).

4 Our approach is similar but not identical to Garicano & Steinwender (2016). Their DiD approach focuses on differences regarding how various types of investment (short-term vs long-term assets) within firms changed following the onset of the crisis, whereas our DiD approach focuses on how various groups of firms (vulnerable vs less vulnerable) changed their self-reported investment following the onset of the crisis.

5 LPMs have previously been applied to difference-in-difference studies that have binary dependent variables (e.g. Bas & Paunov, 2018; Bornhäll et al., 2017). An advantage of applying an LPM in our context is that the estimated coefficients already correspond to marginal effects, and hence the interpretation of LPM estimated coefficients is relatively straightforward, when compared to the output from non-linear models (such as logit regression and conditional logit regression) that require further transformation to obtain estimates of the marginal effects (Angrist & Pischke, 2008).

6 Our set of control variables is therefore similar to Cirera et al. (2021), whose controls are country, sector, size, and time fixed effects.

7 While our main regression analysis focuses on fixed effects regressions, these may perform less well in the context of slowly-changing highly-persistent explanatory variables, and moreover they do not provide coefficient estimates for country dummies. We therefore checked the robustness of our findings using OLS regressions as an alternative to fixed effects regressions, and obtained broadly similar results. Results available from the authors upon request.

8 This is done to maintain consistency across graphs. For example, we do not control for subsidiary firms in these graphs, because this would not be possible in the case where the main explanatory variable is a dummy variable for subsidiary firms; and therefore we prefer to drop subsidiary firms as a control variable in all graphs (even in cases where the main explanatory variable is not the dummy for subsidiary firms).

9 https://ec.europa.eu/info/funding-tenders/find-funding/eu-funding-programmes/recovery-and-resilience-facility_es (last accessed 25 Jan 2022).

10 Seven variables can be mentioned here. First: firms' degrees of digitalization. We have a small number of observations on digitalization in our data, which hinders a detailed analysis. Firms with a higher degree of digitalization could potentially fare better during the COVID crisis, e.g. if their production and sales strategies are more amenable to a sudden shift online in times of lockdown. However, investments in digitalization often display a J-curve, such that there is a long timelag between the time of investment and the actual benefits in terms of enhanced productivity and financial performance (Brynjolfsson et al., 2021). Second: some sectors are more vulnerable than others (Benedetti Fasil et al., 2021; Bloom et al., 2021). An indicator of vulnerable sectors could potentially be constructed (e.g. where vulnerable sectors are defined as sectors with the largest drops in revenue in 2020). Third: some regions/countries could be categorized as more vulnerable, in terms of the extent of liquidity support offered by the government to vulnerable firms. Fourth: future work could potentially have access to indicators of financial vulnerability (*e.g*., firms whose bank loan applications were rejected), to investigate whether financially vulnerable firms fared worse in the COVID crisis. Fifth, exporter firms could be more vulnerable than non-exporters, for example if there are disruptions in distribution networks and supply chains (Meinen et al., 2021). Sixth, firms with high degrees of financial leverage could be vulnerable. Seventh, while we investigate R&D investors as a vulnerable group of firms, nevertheless firms that invest in other intangible assets could be considered to be vulnerable because they may have similar problems regarding issues such as collateral and information asymmetries.

## References

[CR1] Altig D, Baker S, Barrero JM, Bloom N, Bunn P, Chen S, Davis SJ, Leather J, Meyer B, Mihaylov E, Mizen P, Parker N, Renault T, Smietanka P, Thwaites G (2020). Economic uncertainty before and during the COVID-19 pandemic. Journal of Public Economics.

[CR2] Alves, P., Dejuan, D., & Maurin, L. (2019). Can survey-based information help to assess investment gaps in the EU? (No. 2019/04). EIB Working Papers.

[CR3] Angrist JD, Pischke J-S (2008). Mostly harmless econometrics: An empiricist's companion.

[CR4] Balduzzi, P., Brancati, E., Brianti, M., & Schiantarelli, F. (2020). *The Economic Effects of COVID-19 and credit constraints: Evidence from Italian firms’ expectations and plans*. IZA: Institute of Labor Economics, Bonn, Germany. IZA DP No. 13629.

[CR5] Barlevy G (2007). On the cyclicality of research and development. American Economic Review.

[CR6] Bas M, Paunov C (2018). The unequal effect of india's industrial liberalization on firms’ decision to innovate: Do business conditions matter?. Journal of Industrial Economics.

[CR7] Benedetti Fasil, C., Del Rio, J. C., Domnick, C., Fako, P., Flachenecker, F., Gavigan, J., Janiri, M., Stamenov, B. & Testa, G. (2021). High growth enterprises in the COVID-19 crisis context, EUR 30686 EN, Publications Office of the European Union, Luxembourg, 2021. 10.2760/63402, JRC124469.

[CR8] Biancalani F, Czarnitzki D, Riccaboni M (2022). The Italian Start Up Act: A microeconometric program evaluation. Small Business Economics.

[CR9] Bighelli, T., Lalinsky, T., & di Mauro F. (2021). Covid-19 government support may have not been as unproductively distributed as feared. https://voxeu.org/article/covid-19-government-support-may-have-not-been-unproductively-distributed-feared. Accessed 25 Mar 2022

[CR10] Bloom, N., Fletcher, R. S., & Yeh, E. (2021). *The impact of COVID-19 on US firms (No. w28314)*. National Bureau of Economic Research.

[CR11] Bornhäll A, Daunfeldt SO, Rudholm N (2017). Employment protection legislation and firm growth: Evidence from a natural experiment. Industrial and Corporate Change.

[CR12] Brutscher P.-B., Coali A., Delanote J., Harasztosi P. (2020). *EIB Group Survey on Investment and Investment Finance: A technical note on data quality*. European Investment Bank, working paper 2020/08. 10.2867/772584

[CR13] Brynjolfsson E, Rock D, Syverson C (2021). The productivity J-curve: How intangibles complement general purpose technologies. American Economic Journal: Macroeconomics.

[CR14] Cincera M, Veugelers R (2013). Young leading innovators and the EU's R&D intensity gap. Economics of Innovation and New Technology.

[CR15] Cirera X, Cruz M, Davies E, Grover A, Iacovone L, Cordova JEL, Medvedev D, Maduko FO, Nayyar G, Reyes Ortega S, Torres J (2021). Policies to support businesses through the COVID-19 shock: A firm level perspective. World Bank Research Observer.

[CR16] Claeys, G., Darvas, Z., Demertzis, M., & Wolff, G. B. (2021). *The great COVID-19 divergence: managing a sustainable and equitable recovery in the European Union*. Policy Contribution 11/2021, Bruegel, May, 20. 10.1007/s10272-021-0983-8 PMC8339678 34376868

[CR17] Coad A (2018). Firm age: A survey. Journal of Evolutionary Economics.

[CR18] Coad A., Amaral-Garcia S., Bauer P., Domnick C., Harasztosi P., Pal R., Teruel M. (2022a). *High-Growth Enterprises in times of COVID: An overview*. Report prepared for the European Commission, JRC. Unpublished.

[CR19] Coad A, Domnick C, Flachenecker F, Harasztosi P, Janiri ML, Pál R, Teruel M (2021). Capacity constraints as a trigger for high growth. Small Business Economics.

[CR20] Coad A, Frankish JS, Storey DJ (2020). Too fast to live? Effects of growth on survival across the growth distribution. Journal of Small Business Management.

[CR21] Coad A., Harasztosi P., Pál R., Teruel M., (2022b). Policy instruments for high-growth enterprises. In: K. Wennberg, C. Sandström (Eds.) *Chapter 15: Questioning the entrepreneurial state*. Springer Nature. Forthcoming.

[CR22] Cunningham S. (2021). Causal inference: the mixtape. Yale University Press. https://mixtape.scunning.com/index.html

[CR23] Didier T, Huneeus F, Larrain M, Schmukler SL (2021). Financing firms in hibernation during the COVID-19 pandemic. Journal of Financial Stability.

[CR24] Eurostat-OECD (2007). Eurostat-OECD manual on business demography statistics.

[CR25] Flachenecker, F., Gavigan, J., P., Goenaga, X., Pasi, G., Preziosi, N., Stamenov, B., & Testa, G. (2020). *High growth enterprises: Demographics, financing & policy measures*. JRC Technical Report. Joint Research Centre. Brussels, Belgium. https://ec.europa.eu/jrc/en/publication/high-growth-enterprises-demographics-finance-policy-measures

[CR26] Garicano L, Steinwender C (2016). Survive another day: Using changes in the composition of investments to measure the cost of credit constraints. Review of Economics and Statistics.

[CR27] Goodman-Bacon A (2021). Difference-in-differences with variation in treatment timing. Journal of Econometrics, Forthcoming..

[CR28] Gourinchas PO, Kalemli-Özcan Ş, Penciakova V, Sander N (2021). COVID-19 and small-and medium-sized enterprises: A 2021" time bomb"?. American Economic Association Papers and Proceedings.

[CR29] Grassano, N., Hernandez Guevara, H., Fako, P., Tübke, A., Amoroso, S., Georgakaki, A., Napolitano, L., Pasimeni, F., Rentocchini, F., Compaño, R., Fatica, S., & Panzica, R. (2021). *The 2021 EU Industrial R&D Investment Scoreboard*. EUR 30902 EN, Publications Office of the European Union, Luxembourg, JRC127360.

[CR30] Hall BH (2002). The financing of research and development. Oxford Review of Economic Policy.

[CR31] Haltiwanger J, Jarmin RS, Miranda J (2013). Who creates jobs? Small versus Large versus Young. Review of Economics and Statistics.

[CR32] Harasztosi P., Maurin L., Pál R., Revoltella D., van der Wielen W. (2022). *Firm-level policy support during the crisis: So far, so good? European Investment Bank*. EIB Working Paper 2022/01.

[CR33] Helfat CE (2007). Stylized facts, empirical research and theory development in management. Strategic Organization.

[CR34] Huntington-Klein, N. (2021). The effect: An introduction to research design and causality. April 2021. https://nickchk.com/causalitybook.html

[CR35] Khanna T, Yafeh Y (2007). Business groups in emerging markets: Paragons or parasites?. Journal of Economic Literature.

[CR36] Lalinsky, T., & Pál, R. (2021). *Efficiency and effectiveness of the COVID-19 government support: Evidence from firm-level data (No. 2021/06)*. EIB Working Papers.

[CR37] Marques Santos, A., Haegeman, K., and Moncada-Paternò-Castello, P. (2021). *The impact of Covid-19 and of the earlier crisis on firms’ innovation and growth: A comparative analysis*. JRC Working Papers on Territorial Modelling and Analysis No. 03/2021, European Commission, Seville, JRC125490https://ec.europa.eu/jrc/sites/default/files/jrc125490.pdf

[CR38] Meinen, P., Serafini, R., & Papagalli, O. (2021). *Regional economic impact of Covid-19: The role of sectoral structure and trade linkages*. ECB Working Paper Series No 2528, European Central Bank.

[CR39] Mogos S, Davis A, Baptista R (2021). High and sustainable growth: Persistence, volatility, and survival of high growth firms. Eurasian Business Review.

[CR40] Mohnen P, Hall BH (2013). Innovation and productivity: An update. Eurasian Business Review.

[CR41] Moncada-Paternò-Castello P (2022). Top R&D investors, structural change and the R&D growth performance of young and old firms. Eurasian Business Review.

[CR42] Pellegrino G, Piva M (2020). Innovation, industry and firm age: Are there new knowledge production functions?. Eurasian Business Review.

[CR43] Piva M, Vivarelli M (2007). Is demand-pulled innovation equally important in different groups of firms?. Cambridge Journal of Economics.

[CR44] Roper S, Turner J (2020). R&D and innovation after COVID-19: What can we expect? A review of prior research and data trends after the great financial crisis. International Small Business Journal.

[CR45] Rostamkalaei A, Freel M (2016). The cost of growth: Small firms and the pricing of bank loans. Small Business Economics.

[CR46] Roth, J., Sant'Anna, P. H., Bilinski, A., & Poe, J. (2022). *What's trending in difference-in-differences? A synthesis of the recent econometrics literature*. arXiv preprint arXiv:2201.01194.

[CR47] Storey DJ (1994). Understanding the small business sector.

[CR48] Vivarelli M (2016). The middle income trap: A way out based on technological and structural change. Economic Change and Restructuring.

[CR49] Zhang, M., & Mohnen, P. (2022). R&D, innovation and firm survival in Chinese manufacturing, 2000–2006. *Eurasian Business Review,* 1–37.

