# Supplementary material for: Investment expectations by vulnerable European firms in times of COVID
Source: Eurasian Bus Rev. 2022 Aug 10;13(1):193–220. doi: 10.1007/s40821-022-00218-z (PMC9364289; doi:10.1007/s40821-022-00218-z)
Supplement: Supplementary file 1 — Supplementary file1 (DOCX 2023 kb) [file 40821_2022_218_MOESM1_ESM.docx]

ONLINE SUPPLEMENTARY MATERIALS

APPENDIX OSM 1: event study graphs.

Datapoints are obtained from regressions that include control variables (sector and country dummies), using robust standard errors.

Graphs are organized in the following way:

- By group of vulnerable firm: HGE, subsidiary, R&D investor, and also the interaction term young × small.
- Then, in the order shown in the table below (by questions: q21_n; q21_p; q23_1_n; q23_1_p; q23_2_n; q23_2_p; q23_3_n; q23_3_p; q23_4_n; q23_4_p; q23_5_n; q23_5_p; q25_1; q25_2; q25_3)

Appendix Table OSM-1: Information on the survey questions regarding investment expectations

| Survey question | Summary | Wording of the survey question |
| --- | --- | --- |
| q21 | Expected change in investment | For the current financial year, do you expect your total investment spend to be: A. More than last year; B. Around the same amount as last year; C. Less than last year; D. No investment planned |
| q23_1 | Availability of internal finance | Do you think that each of the following will improve, stay the same, or get worse over the next 12 months? A. Availability of internal finance within the company (e.g. internal funds like cash). Possible answers: Improve; Stay the same; Deteriorate |
| q23_2 | Availability of external finance | Do you think that each of the following will improve, stay the same, or get worse over the next 12 months? B. Availability of external finance (e.g. bank financing, private or public equity). Possible answers: Improve; Stay the same; Deteriorate |
| q23_3 | Industry’s business prospects | Do you think that each of the following will improve, stay the same, or get worse over the next 12 months? C. Business prospects specific to your sector or industry. Possible answers: Improve; Stay the same; Deteriorate |
| q23_4 | Overall economic climate | Do you think that each of the following will improve, stay the same, or get worse over the next 12 months? D. Overall economic climate. Possible answers: Improve; Stay the same; Deteriorate |
| q23_5 | Political & regulatory climate | Do you think that each of the following will improve, stay the same, or get worse over the next 12 months? E. Political and regulatory climate. Possible answers: Improve; Stay the same; Deteriorate |
| q25_1 | New products/processes | Investment priority in the next three years: A. Developing or introducing new products, processes or services |
| q25_2 | Replacing capacity | Investment priority in the next three years: B. Replacing capacity (including existing buildings, machinery, equipment and IT) |
| q25_3 | Capacity expansion | Investment priority in the next three years: C. Capacity expansion for existing products/services |

Source: EIBIS survey.

**HGEs**


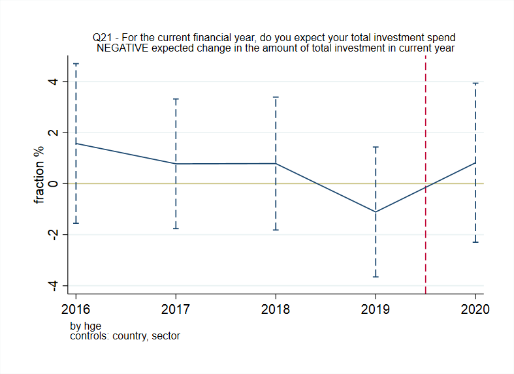

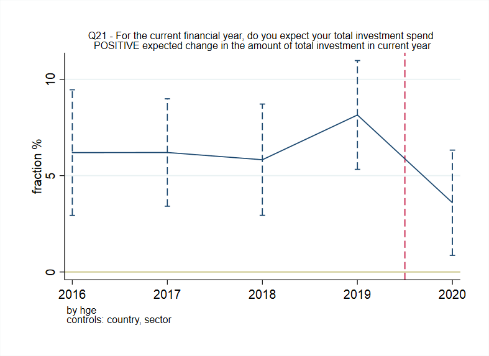


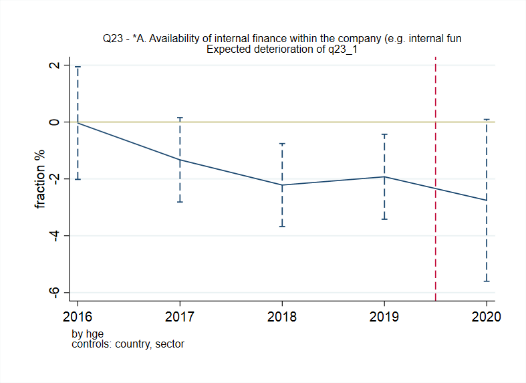

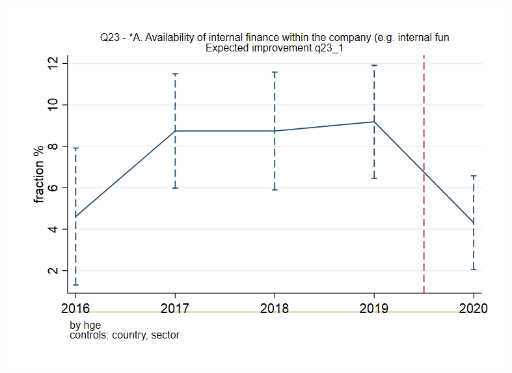


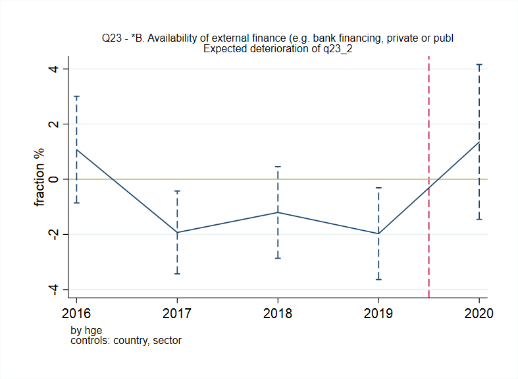

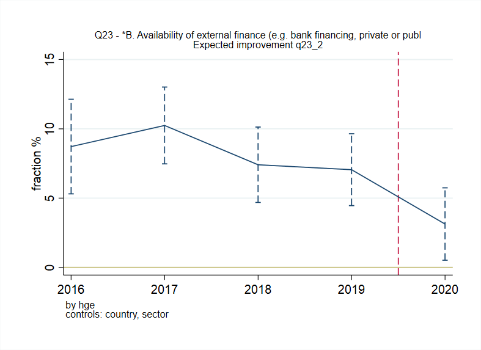


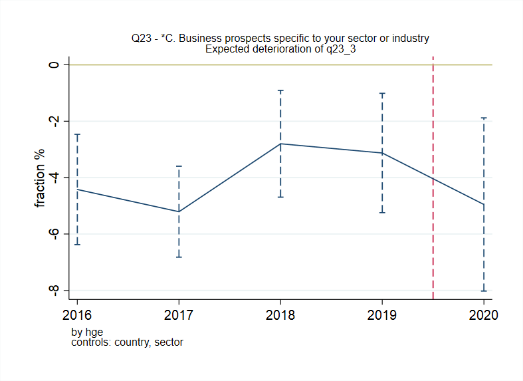

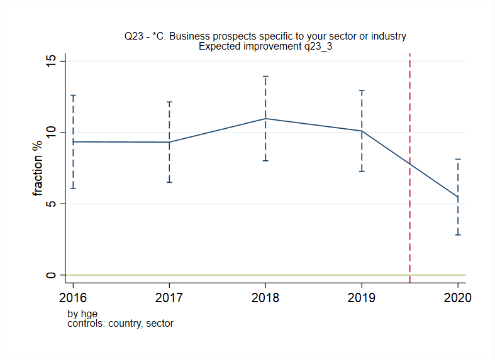


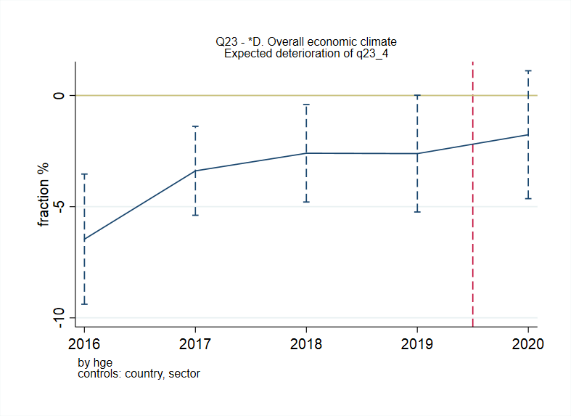

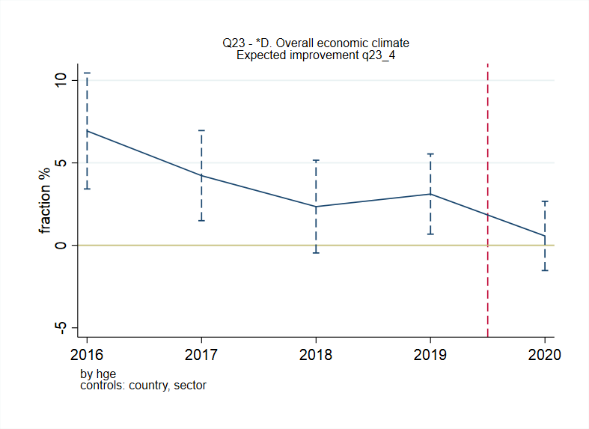


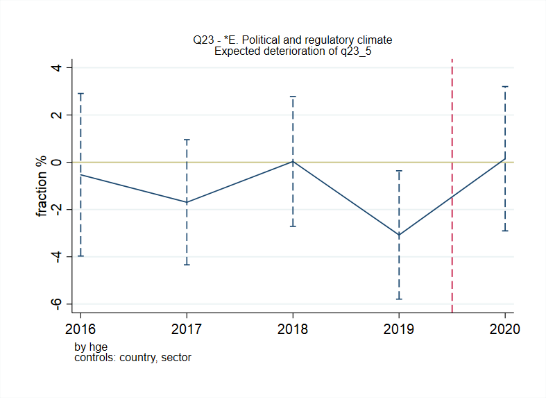

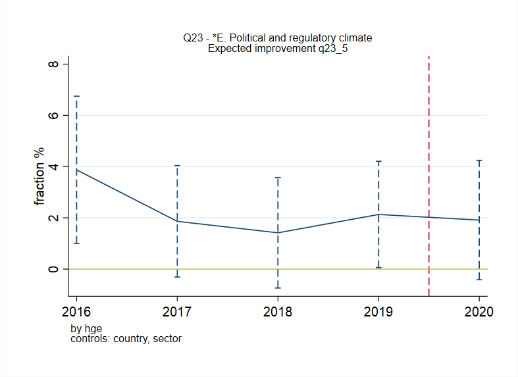


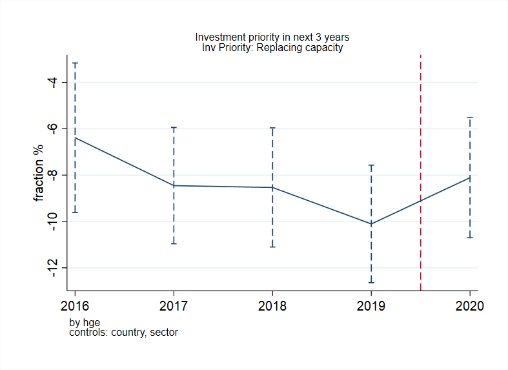

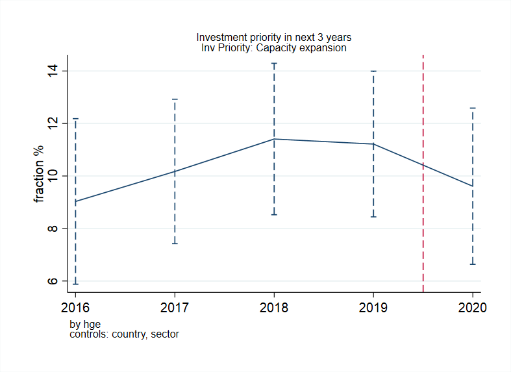


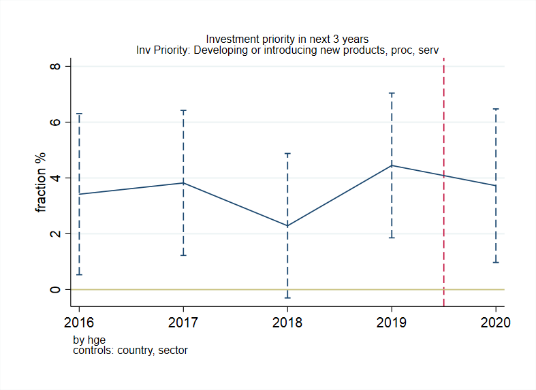


**SUBSIDIARY**


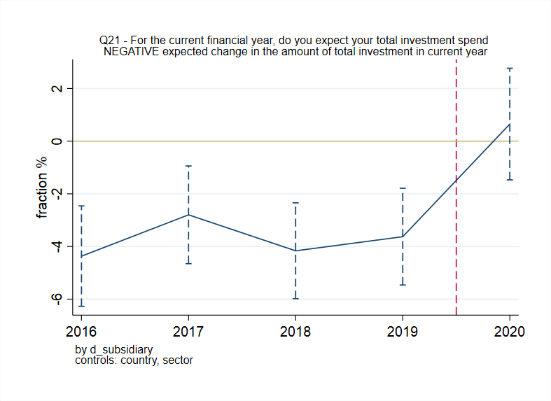

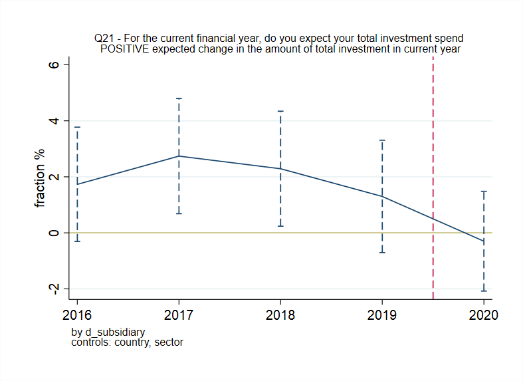


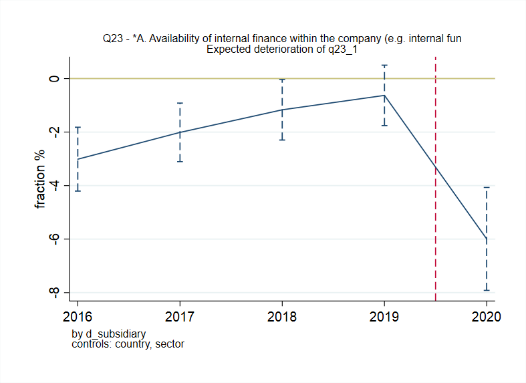

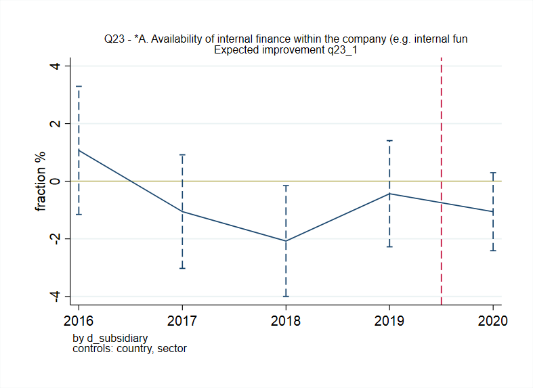


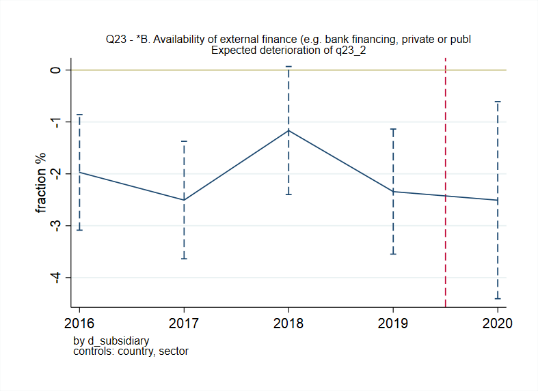

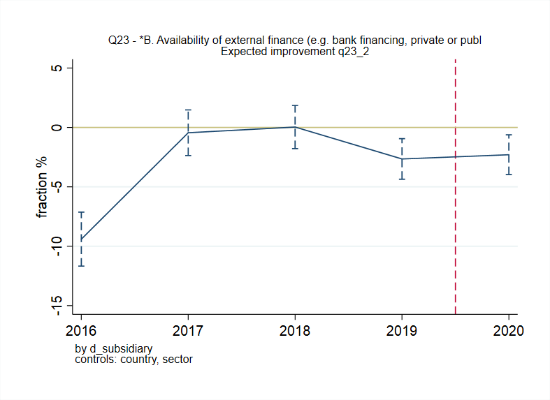


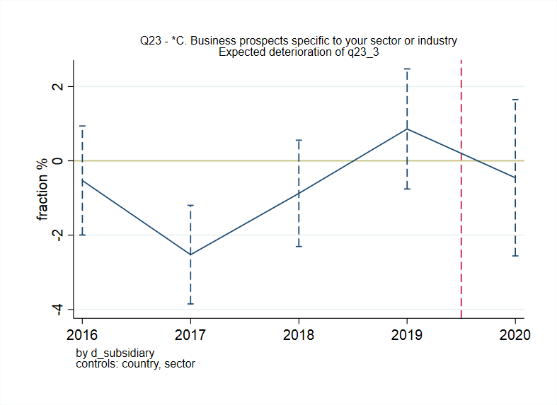

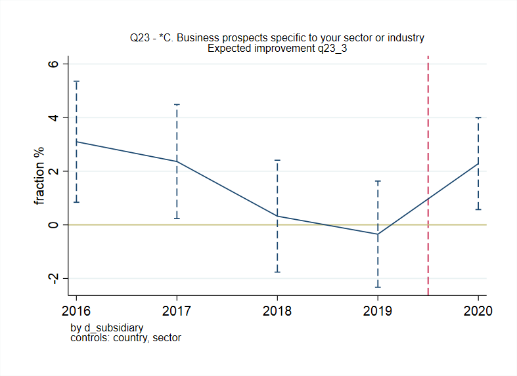


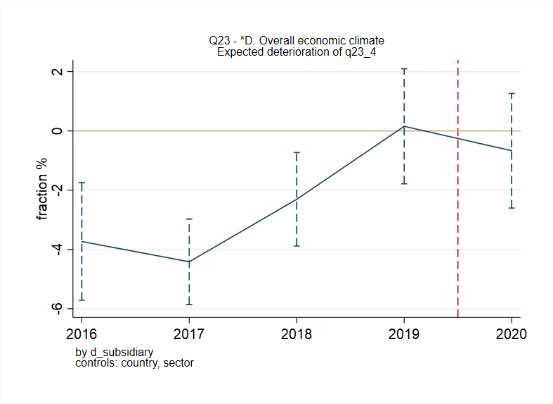

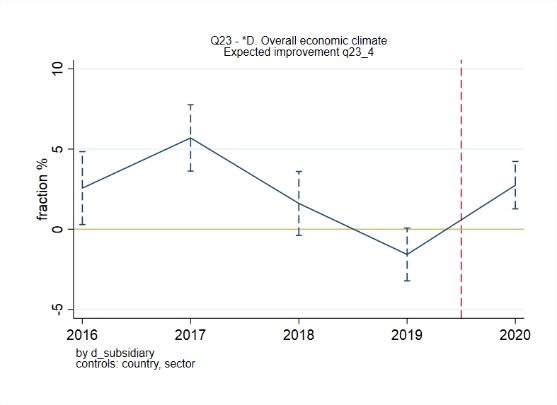


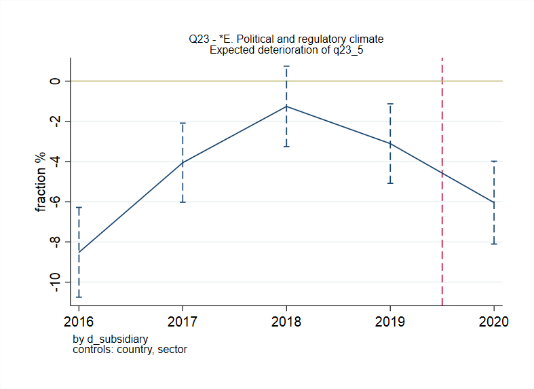

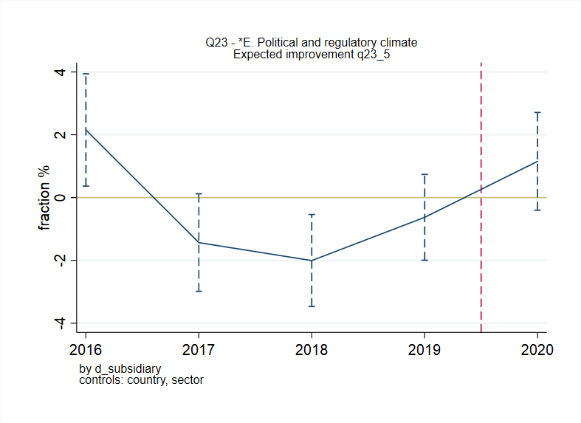


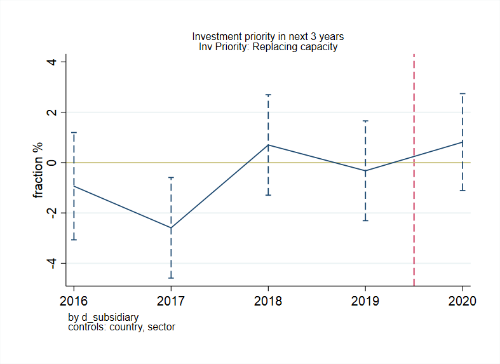

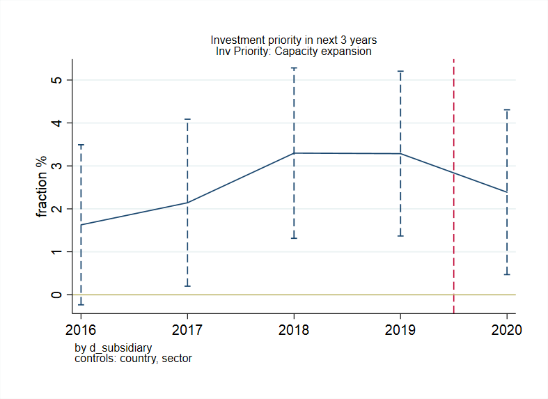


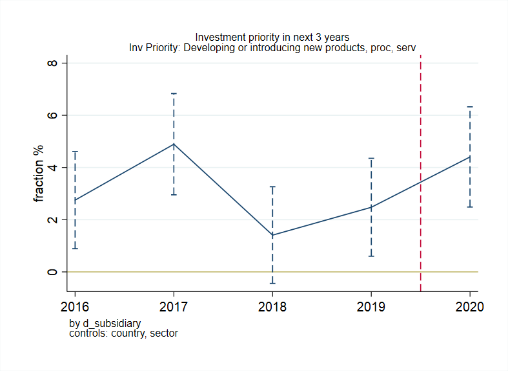


**R&D INVESTORS**


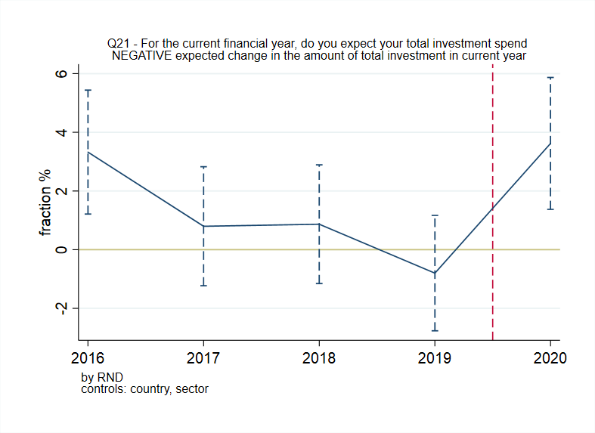

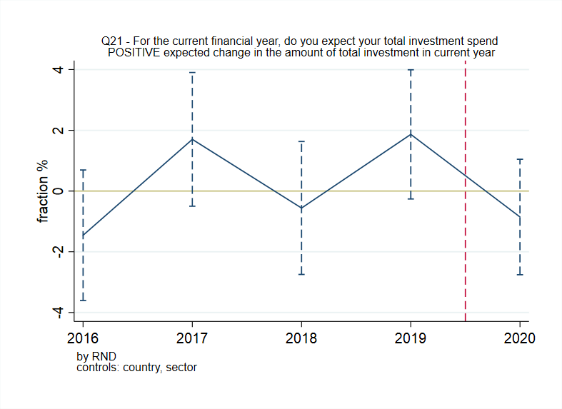


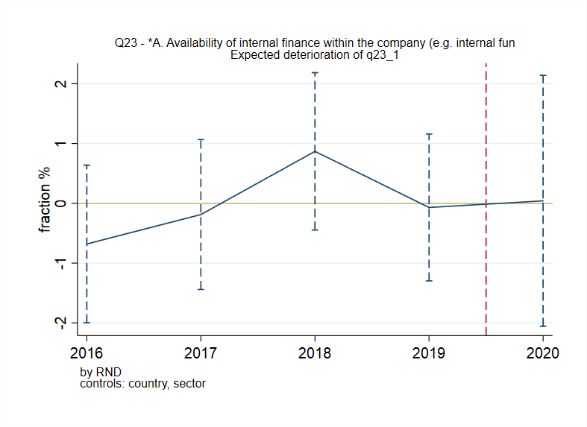

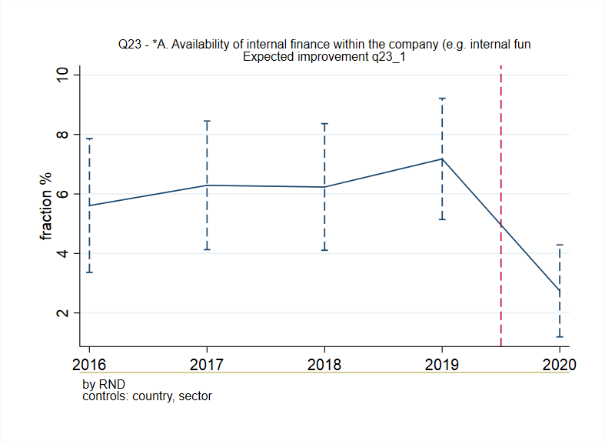


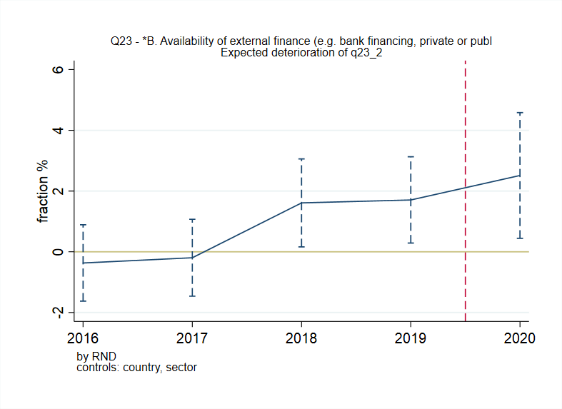

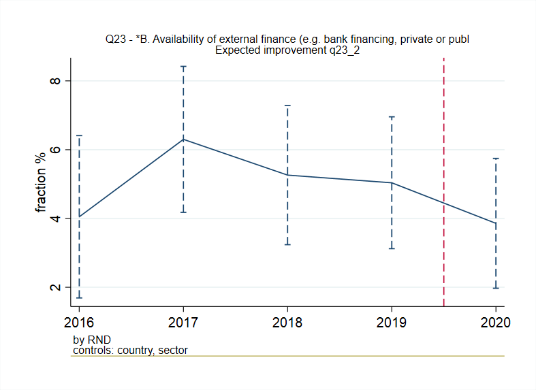


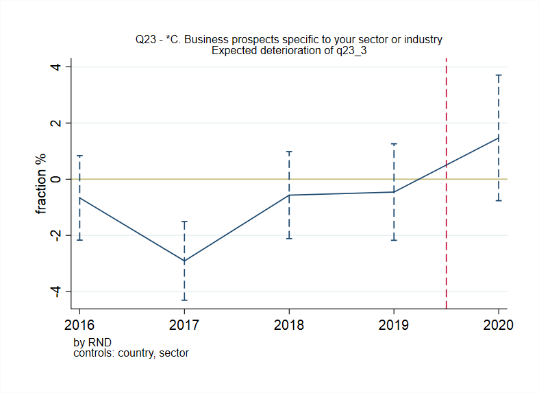

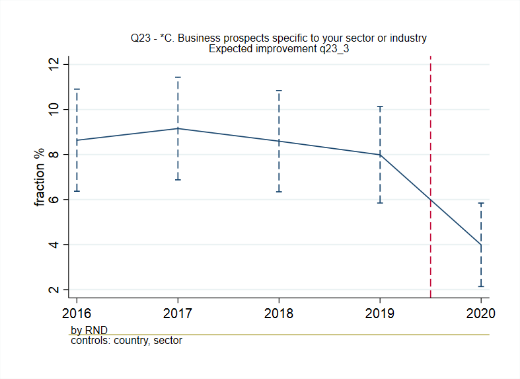


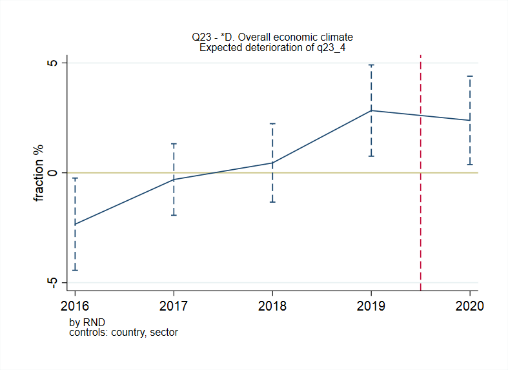

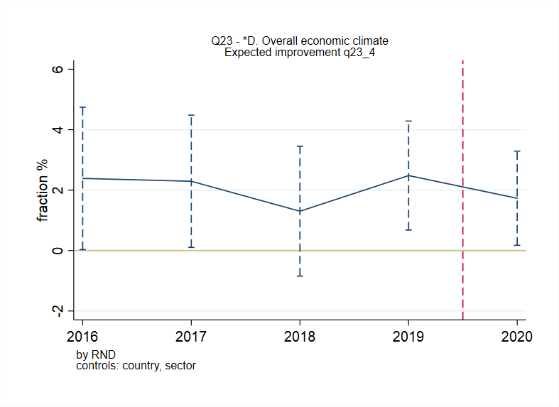


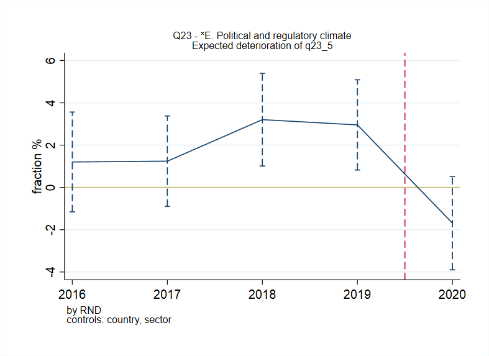

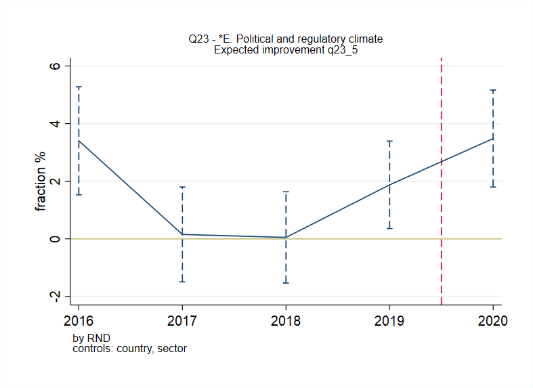


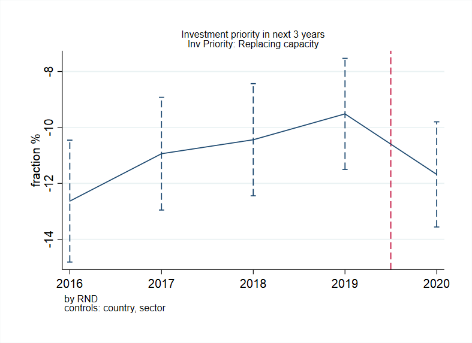

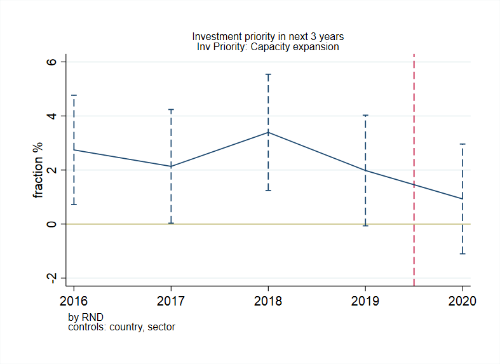


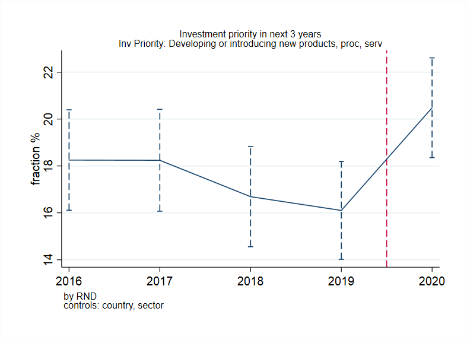


**INTERACTION TERM: YOUNG × SMALL FIRMS**


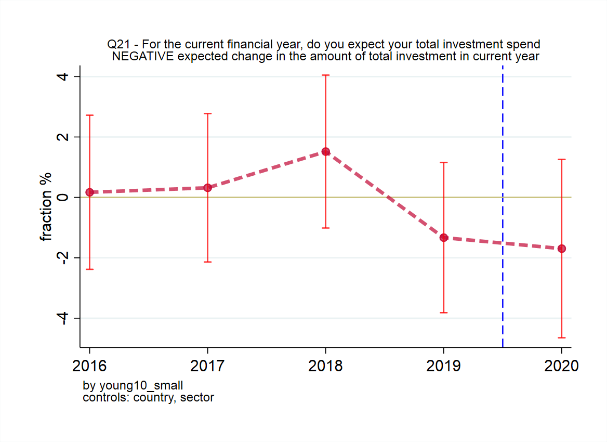

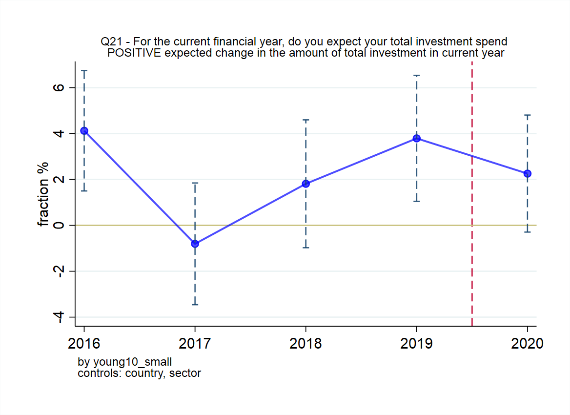


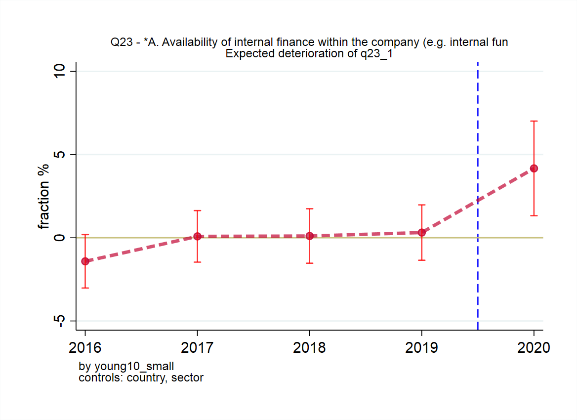

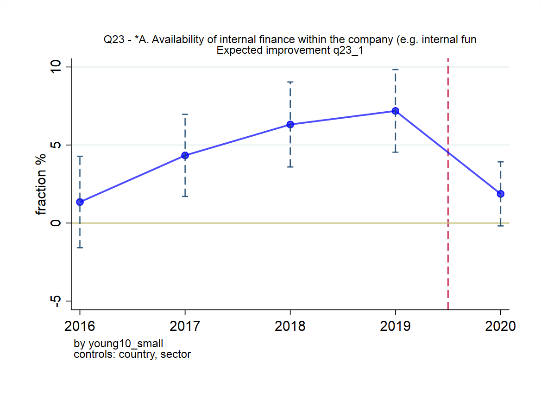


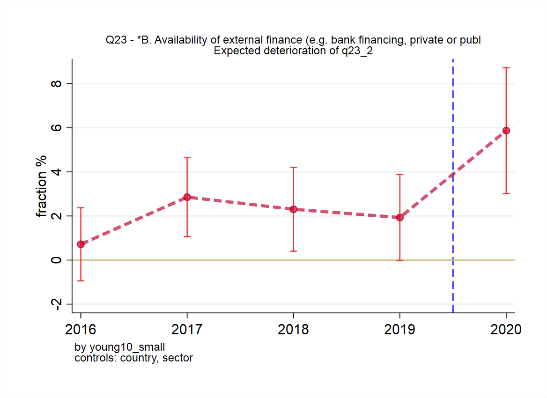

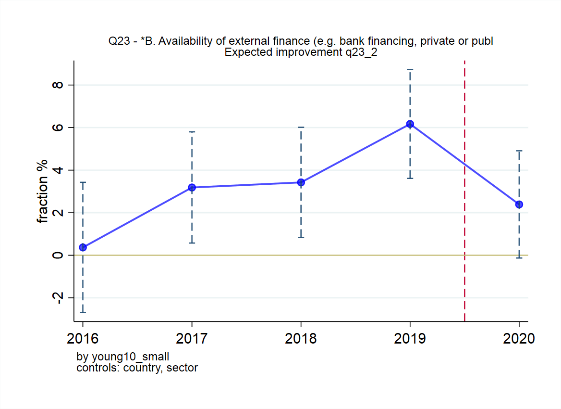


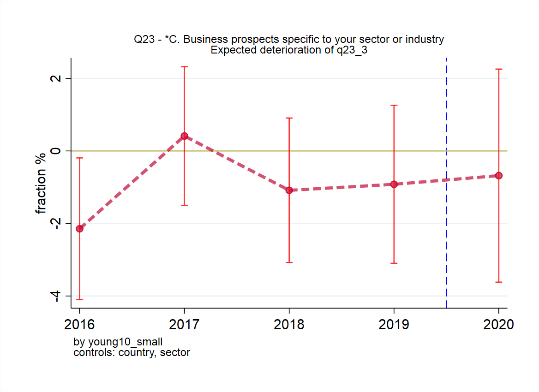

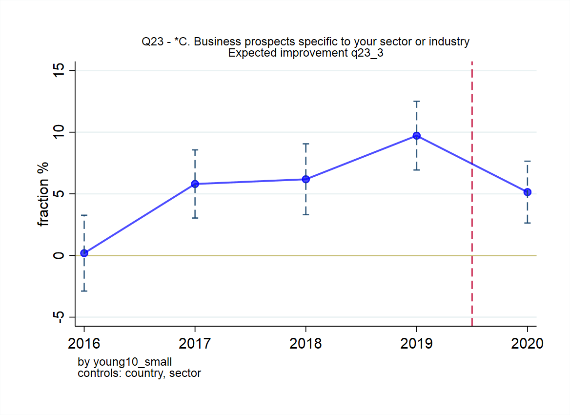


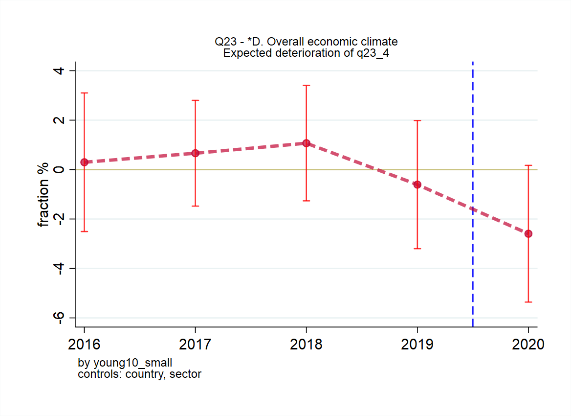

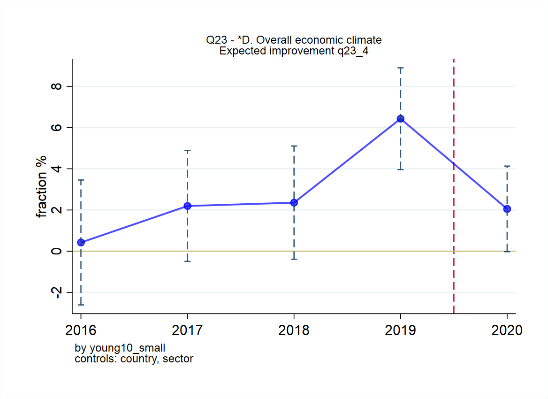


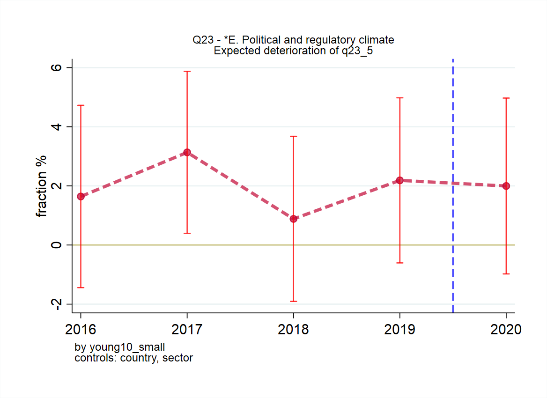

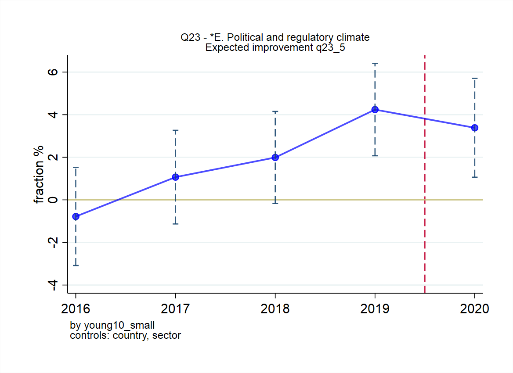


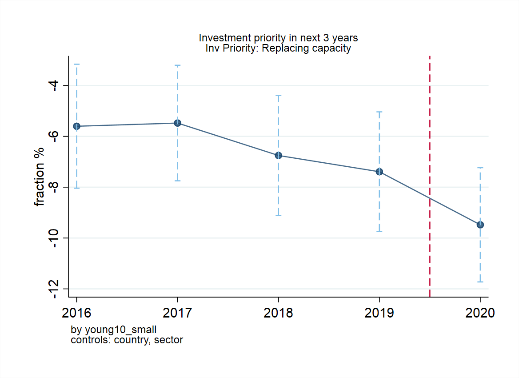

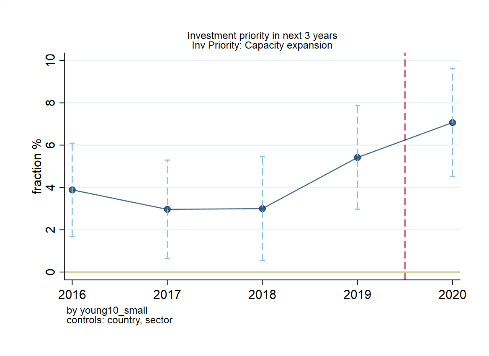


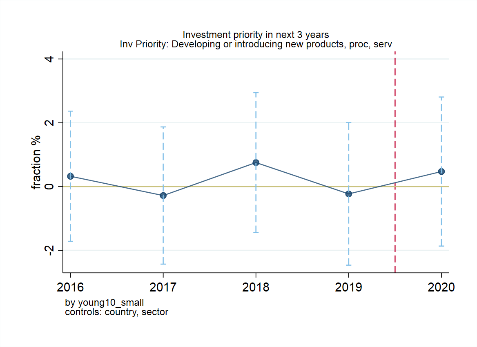


APPENDIX OSM 2: business environment, economic climate, and investment priorities

Table OSM-2.1 shows various results for the expectations of vulnerable firms regarding their industry's business prospects, the overall economic climate, and the political and regulatory climate. HGEs are observed to be less likely to have positive expectations about their industry’s business prospects and about the overall economic climate.

R&D investors tend to have a pessimistic view on their industry’s business prospects, in the sense that they are significantly less likely to report positive expectations. This could be a problem, because theoretical work has emphasized that R&D investment depends on optimistic projections about the state of the economy and regarding the size of the market (and hence demand for the R&D outputs) (Barlevy, 2007; Piva and Vivarelli, 2007).

Regarding subsidiary firms, which are a case of non-vulnerable firms, Table OSM-2.1 suggests that overall they are quite optimistic. The only significant coefficient in their case suggests that they are more likely to report positive expectations regarding their industry’s business prospects.

Table OSM-2.1: Estimates of the DiD coefficient of $\beta_{1}$ obtained from FE (i.e. within) regressions of equation (1). Controls include country × year and sector × year fixed effects. This table summarizes results from 6 × 4 = 24 different regressions (i.e. 6 alternative dependent variables and 4 alternative proxies for “vulnerable” firms). The table shows coefficients as well as t-statistics (in parentheses) that are obtained after clustering the standard errors at the firm level. Control variables, constant term, and model fit statistics for the regressions are not shown here for conciseness.

|  | HGE | subsidiary | R&D | young × small |
| --- | --- | --- | --- | --- |
| Industry’s business prospects: negative | -0.405 | 0.357 | 2.395 | -2.406 |
|  | (-0.16) | (0.21) | (1.28) | (-0.97) |
| Industry’s business prospects: positive | **-6.419** | **3.767** | **-4.042** | -1.247 |
|  | (-2.38) | (2.22) | (-2.20) | (-0.5) |
| Overall economic climate: negative | 0.489 | 0.759 | 1.357 | **-6.696** |
|  | (0.18) | (0.44) | (0.73) | (-2.72) |
| Overall economic climate: positive | -3.004 | 2.828 | -0.250 | 1.540 |
|  | (-1.27) | (1.90) | (-0.15) | (0.73) |
| Political & regulatory climate: negative | 1.998 | 0.144 | -1.081 | -1.995 |
|  | (0.69) | (0.08) | (-0.55) | (-0.75) |
| Political & regulatory climate: positive | -1.536 | 1.708 | 0.752 | 4.137 |
|  | (-0.64) | (1.20) | (0.47) | (1.88) |

Source: EIBIS survey, our analysis. Notes: coefficients significant at the 5% level appear in bold.

Here we investigate responses regarding the survey question on business prospects specific to the sector or industry.

Figure OSM-2.1: Responses regarding business prospects and overall economic climate. Top: HGEs, expected improvement. Bottom Left: R&D investors, expected improvement. Bottom Right: subsidiary firms, expected improvement.


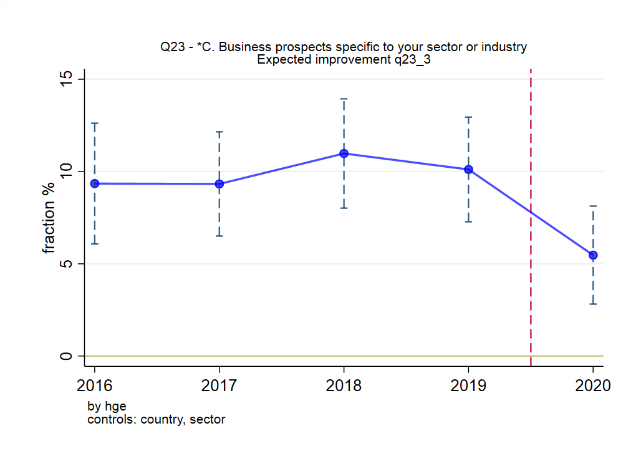


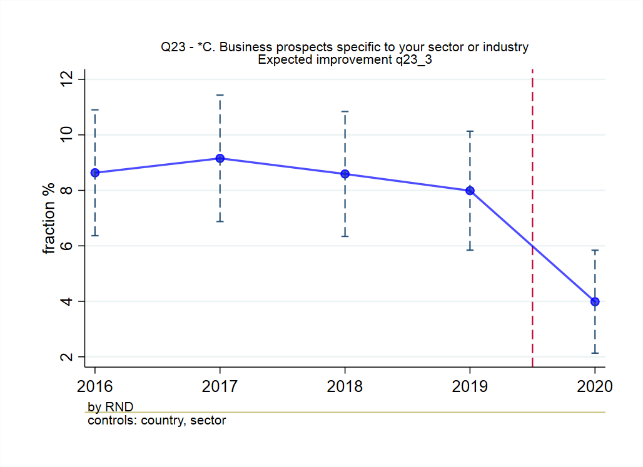

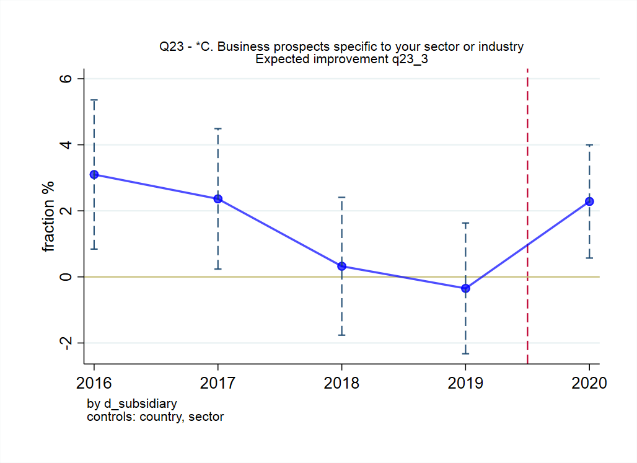


Source: EIBIS survey, our analysis. NOTES: Datapoints obtained from regressions that include control variables (sector and country dummies), using robust standard errors.

Figure OSM-2.1 shows that subsidiaries are more likely to report an improvement in business prospects (Figure OSM-2.1, bottom right). Perhaps, subsidiaries have business opportunities that will allow them to thrive despite the challenges brought on by COVID, for example opportunities brought on by digitalization and the reorganization of business processes and economic activity.

Figure OSM-2.1 also shows that some categories of firms are less optimistic about business prospects after COVID. In particular, HGEs (Figure OSM-2.1, top) and R&D investors (Figure OSM-2.1, bottom left) are somewhat less likely to expect an improvement in business prospects in the 2020 COVID wave.

Table OSM-2.2 presents the results from the survey questions on investment priorities, which correspond to three areas: developing or introducing new products, processes or services; replacing capacity, and capacity expansion. No significant results are found for HGEs, young and small firms, subsidiaries, or R&D investors. Both HGEs and R&D investors report putting less emphasis on replacement, and more emphasis on introducing new products, and services, although these results did not attain statistical significance.

Table OSM-2.2: Estimates of the DiD coefficient of $\beta_{1}$ obtained from FE (i.e. within) regressions of equation (1). Controls include country × year and sector × year fixed effects. This table summarizes results from 3 × 4 = 12 different regressions (i.e. 3 alternative dependent variables and 4 alternative proxies for “vulnerable” firms). The table shows coefficients as well as t-statistics that are obtained after clustering the standard errors at the firm level. Control variables, constant term, and model fit statistics for the regressions are not shown here for conciseness.

|  | HGE | subsidiary | R&D | young × small |
| --- | --- | --- | --- | --- |
| Replacing capacity | 3.127 | 0.739 | 0.980 | -2.765 |
|  | (1.26) | (0.44) | (0.57) | (-1.19) |
| Capacity expansion | -3.172 | 0.038 | -2.135 | 3.726 |
|  | (-1.13) | (0.02) | (-1.19) | (1.53) |
| New products/processes | 0.327 | 2.801 | 2.725 | -2.634 |
|  | (0.13) | (1.76) | (1.51) | (-1.24) |

Source: EIBIS survey, our analysis. Notes: coefficients significant at the 5% level appear in bold.

We also inspect the event study graphs regarding investment priorities. Figure OSM-2.2 shows the investment priorities of R&D investors following on from the COVID outbreak. R&D investors report putting less emphasis on replacement capacity,^[[1]](#footnote-1)^ and more emphasis on introducing new products, processes, and services. Similar results for R&D investors were found in the regression results in Table OSM-2.2, although they did not attain statistical significance.

Figure OSM-2.2: Responses regarding (different areas of) investment priorities. Left: R&D investors, replacing capacity. Right: R&D investors, developing new products & processes.


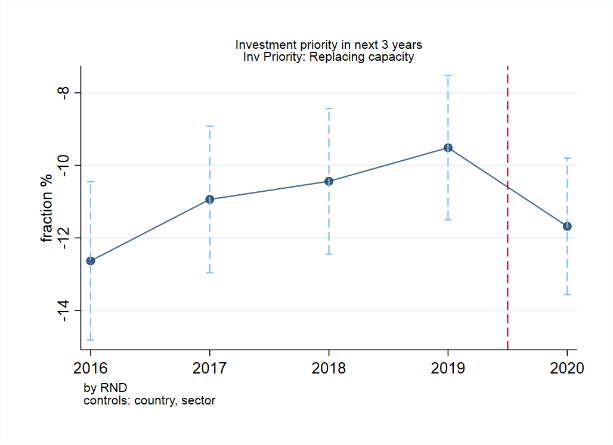

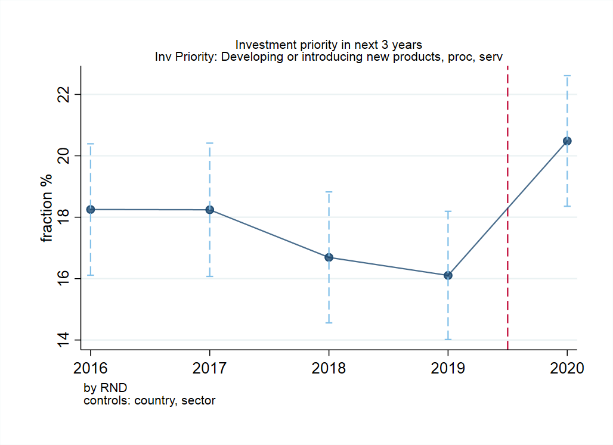


Source: EIBIS survey, our analysis. NOTES: Datapoints obtained from regressions that include control variables (sector and country dummies), using robust standard errors.

APPENDIX OSM 3: descriptive statistics for the investment expectations of HGEs, 2019-2020

Table OSM-3.1: Proportions of firms corresponding to different responses regarding expected total investment spend in the current financial year.

|  | Non-HGEs | | HGEs | |
| --- | --- | --- | --- | --- |
|  | 2019 | 2020 | 2019 | 2020 |
| A. More than last year | 29% | 19% | 39% | 25% |
| B. Around the same amount as last year | 43% | 24% | 38% | 23% |
| C. Less than last year | 22% | 47% | 20% | 42% |
| D. No investment planned | 5% | 9% | 3% | 7% |

Source: EIBIS survey, our analysis. Notes: column totals calculated after removing “Refused” and “Don’t Know.” Value added weights are applied.

APPENDIX OSM 4: sectoral drivers of the COVID impact

Figure OSM-4.1: Sales decline and share of firms with expected decrease of investments.


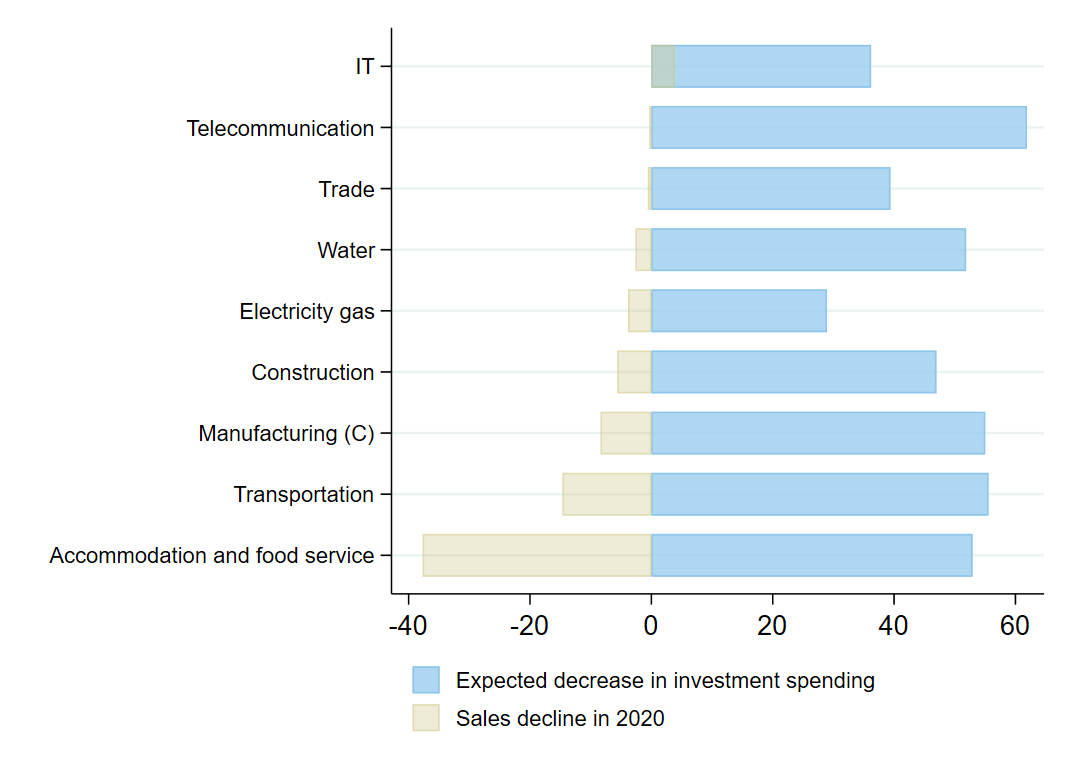


Figure OSM-4.2: Sales decline and share of firms with expected increase of investments.


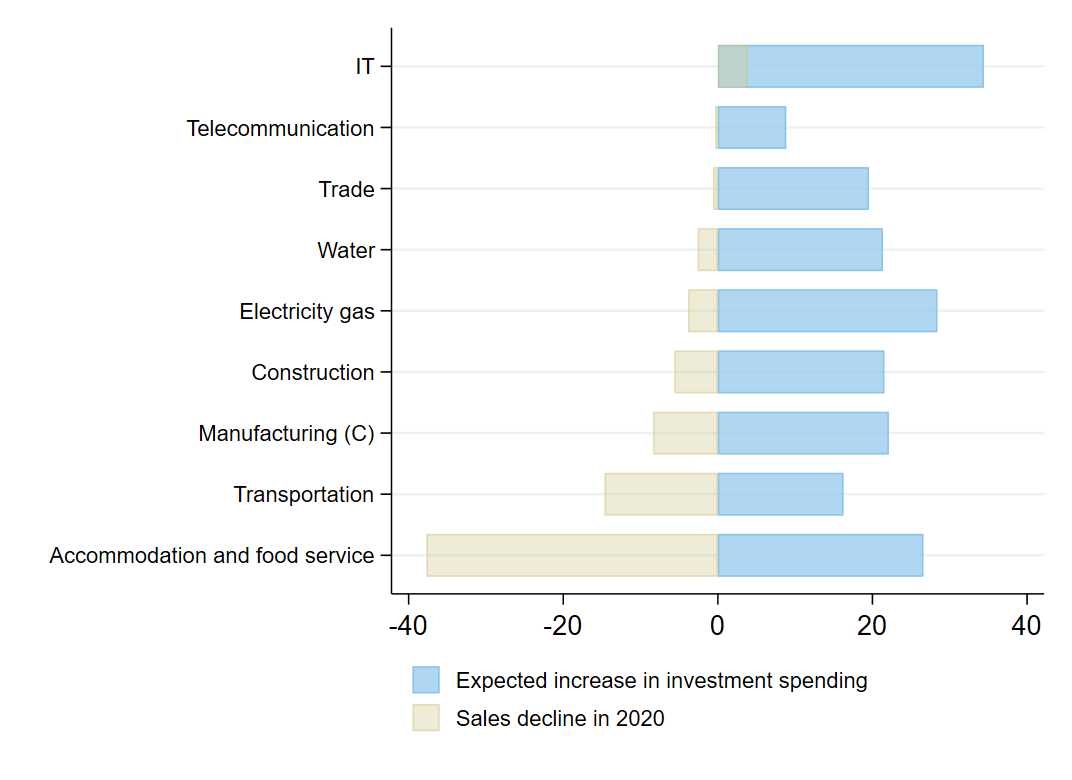


Figure OSM-4.3: Sales decline and share of firms with expected deterioration of the industry business prospects


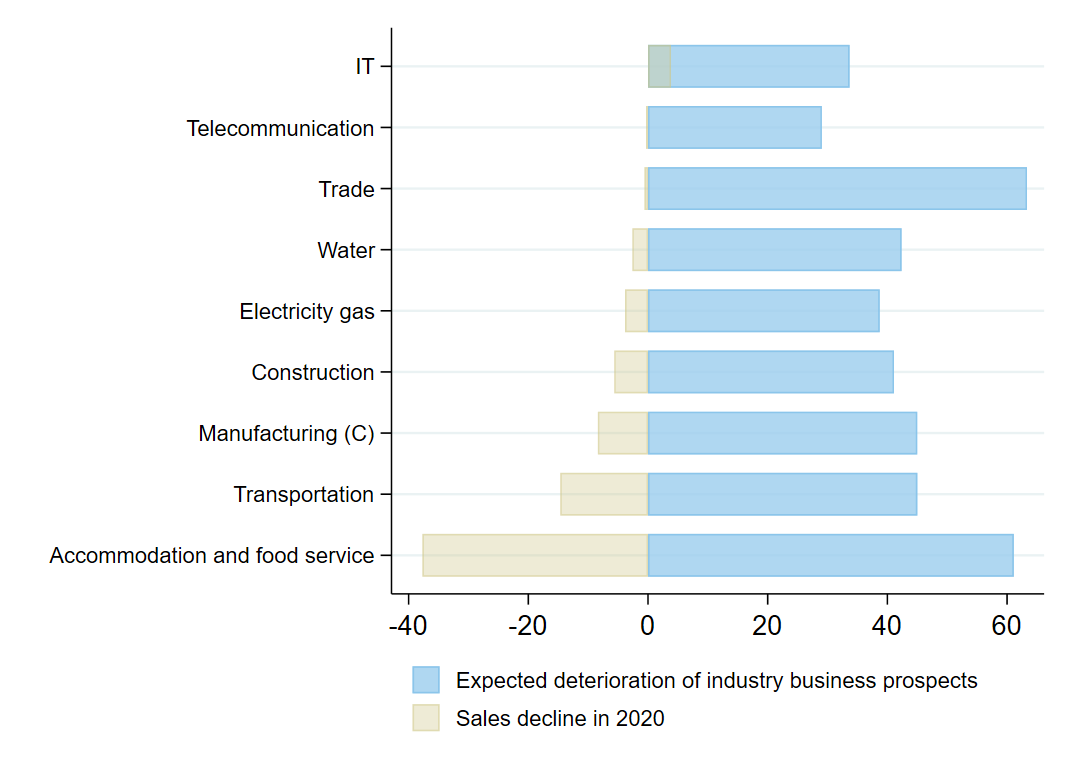


Figure OSM-4.4: Sales decline and share of firms with expected improvement of the industry business prospects


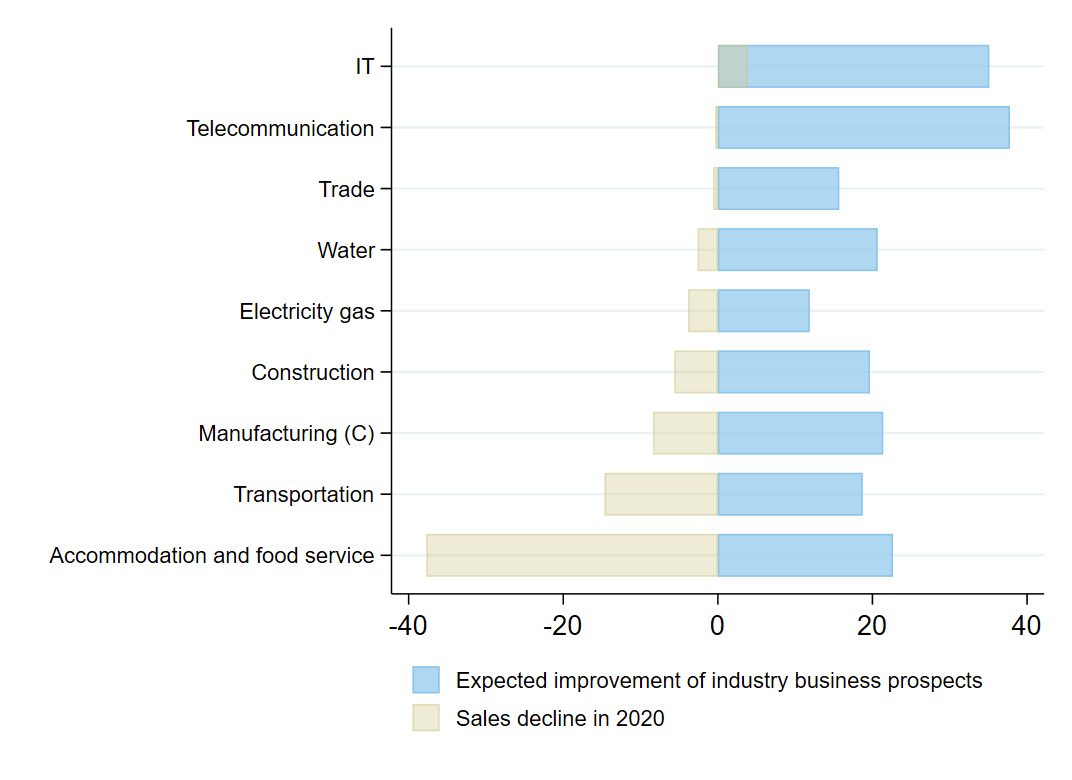


Figure OSM-4.5: Sales decline and share of firms considering replacement capacity as an investment priority


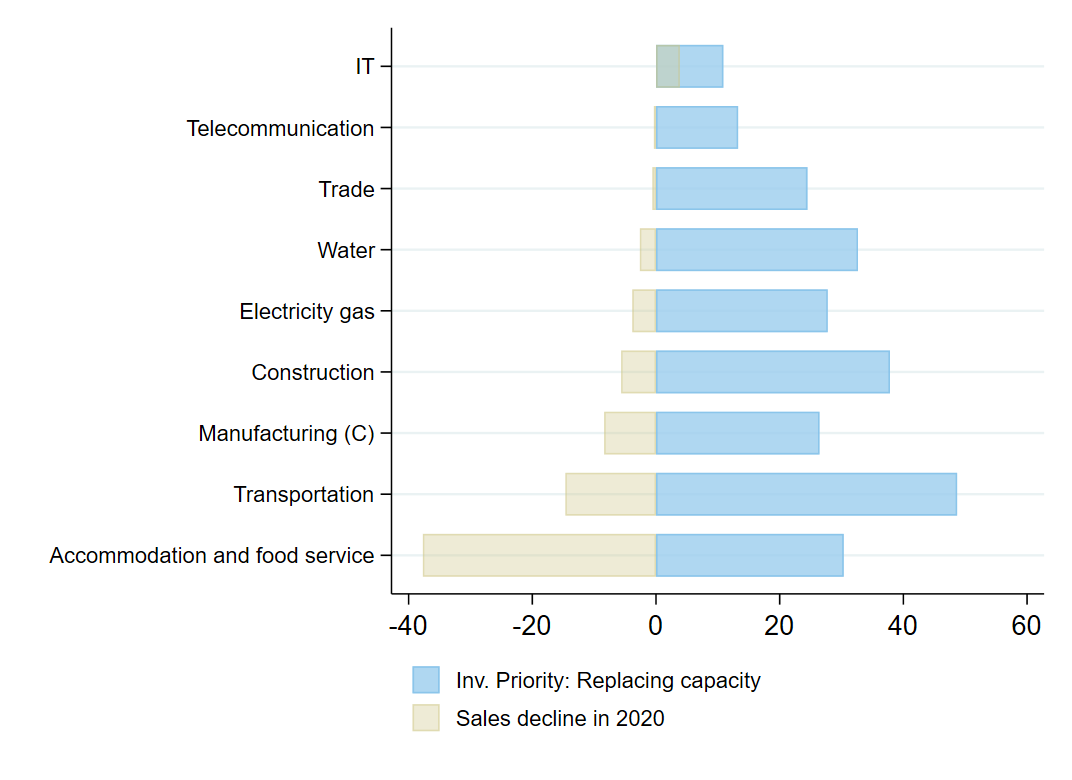


Figure OSM-4.6: Sales decline and share of firms considering capacity expansion as an investment priority


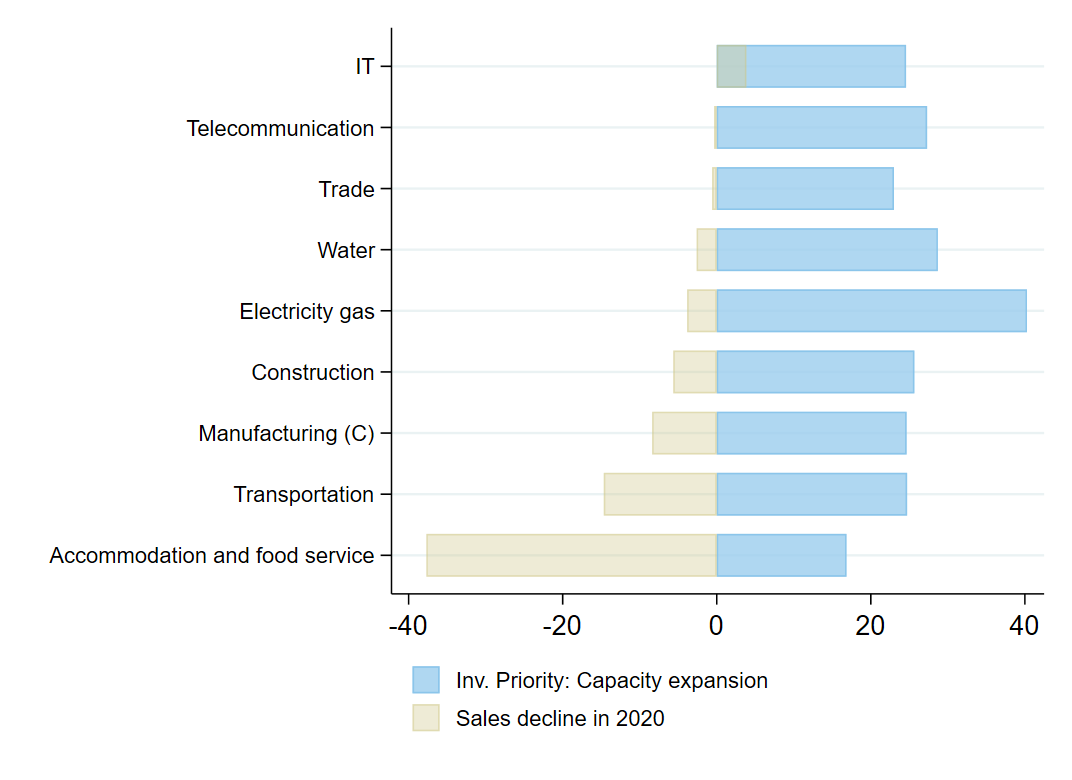


Figure OSM-4.7: Sales decline and share of firms considering new products and services as an investment priority


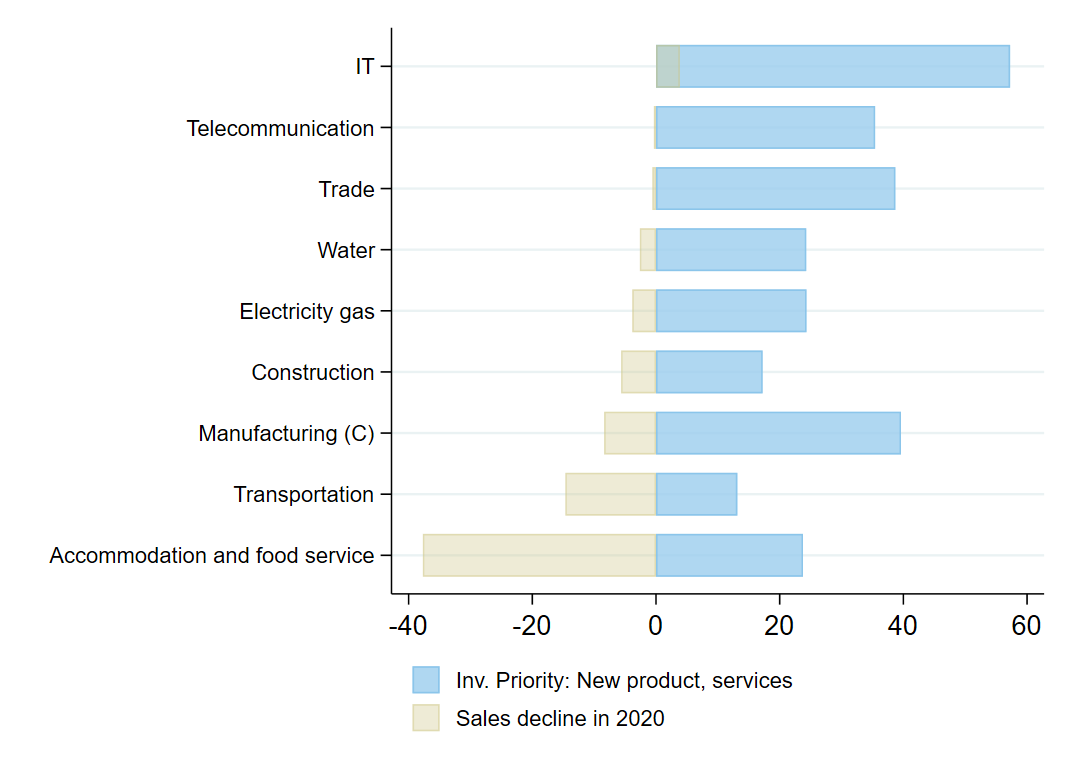


APPENDIX OSM 5: summary statistics

Table OSM-5.1: Countries in our sample (for the 2020 wave)

| Country_ISO_code | Freq. | Percent | Cum. |
| --- | --- | --- | --- |
| AT | 480 | 3.82 | 3.82 |
| BE | 480 | 3.82 | 7.64 |
| BG | 480 | 3.82 | 11.45 |
| CY | 180 | 1.43 | 12.89 |
| CZ | 481 | 3.83 | 16.71 |
| DE | 601 | 4.78 | 21.49 |
| DK | 480 | 3.82 | 25.31 |
| EE | 400 | 3.18 | 28.49 |
| ES | 600 | 4.77 | 33.26 |
| FI | 480 | 3.82 | 37.08 |
| FR | 601 | 4.78 | 41.86 |
| GB | 601 | 4.78 | 46.64 |
| GR | 403 | 3.21 | 49.85 |
| HR | 488 | 3.88 | 53.73 |
| HU | 481 | 3.83 | 57.56 |
| IE | 401 | 3.19 | 60.75 |
| IT | 601 | 4.78 | 65.53 |
| LT | 400 | 3.18 | 68.71 |
| LU | 180 | 1.43 | 70.14 |
| LV | 370 | 2.94 | 73.08 |
| MT | 180 | 1.43 | 74.51 |
| NL | 480 | 3.82 | 78.33 |
| PL | 483 | 3.84 | 82.17 |
| PT | 481 | 3.83 | 86 |
| RO | 480 | 3.82 | 89.82 |
| SE | 480 | 3.82 | 93.64 |
| SI | 400 | 3.18 | 96.82 |
| SK | 400 | 3.18 | 100 |
| Total | 12,572 | 100 |  |

Table OSM-5.2: Sector decomposition for our sample (for the 2020 wave)

| Company Sector | Freq. | Percent | Cum. |
| --- | --- | --- | --- |
| Manufacturing | 3,746 | 30 | 29.83 |
| Construction | 2,689 | 21 | 51.25 |
| Services | 3,243 | 26 | 77.08 |
| Infrastructure | 2,878 | 22.92 | 100 |
| Total | 12,556 | 100 |  |

Table OSM-5.3: descriptive statistics for the investment-related dependent variables, for the 2020 wave

| **Variable** | **Description** | **Obs** | **Mean** | **Std. dev.** | **Min** | **Max** |
| --- | --- | --- | --- | --- | --- | --- |
| q21_bal_p | POSITIVE expected change in the amount of total investment in current year | 12,355 | 0.231809 | 0.4220047 | 0 | 1 |
| q21_bal_n | NEGATIVE expected change in the amount of total investment in current year | 12,355 | 0.4865237 | 0.4998386 | 0 | 1 |
| q23_1_bal_p | Expected improvement of availability of internal finance | 12,296 | 0.120039 | 0.3250204 | 0 | 1 |
| q23_1_bal_n | Expected deterioration of availability of internal finance | 12,296 | 0.3306766 | 0.4704759 | 0 | 1 |
| q23_2_bal_p | Expected improvement of availability of external finance | 11,688 | 0.2032854 | 0.4024603 | 0 | 1 |
| q23_2_bal_n | Expected deterioration of availability of external finance | 11,688 | 0.280373 | 0.4492007 | 0 | 1 |
| q23_3_bal_p | Expected improvement of sector/industry business prospects | 12,174 | 0.1908165 | 0.3929609 | 0 | 1 |
| q23_3_bal_n | Expected deterioration of sector/industry business prospects | 12,174 | 0.4513718 | 0.4976501 | 0 | 1 |
| q23_4_bal_p | Expected improvement of overall economic climate | 12,238 | 0.1260827 | 0.3319561 | 0 | 1 |
| q23_4_bal_n | Expected deterioration of overall economic climate | 12,238 | 0.7051806 | 0.4559802 | 0 | 1 |
| q23_5_bal_p | Expected improvement of political and regulatory climate | 11,580 | 0.1450777 | 0.3521944 | 0 | 1 |
| q23_5_bal_n | Expected deterioration of political and regulatory climate | 11,580 | 0.403886 | 0.4906963 | 0 | 1 |

1. Note however that this negative coefficient for 2020 for R&D investors for expected change in total investment is not a clear break from the values taken in previous years, and the corresponding regression coefficients in Tables 3 and 4 are not statistically significant. Stronger, however, is the result for 2020 for R&D investors for expected change in overall economic climate. [↑](#footnote-ref-1)
